# Prothrombin complex concentrate for reversal of oral anticoagulants in patients with oral anticoagulation-related critical bleeding: a systematic review of randomised clinical trials

**DOI:** 10.1186/s13049-025-01334-1

**Published:** 2025-02-04

**Authors:** Christian Ovesen, Jan Purrucker, Josefine Grundtvig, Theis Bech Mikkelsen, Christian Gluud, Janus Christian Jakobsen, Hanne Christensen, Thorsten Steiner

**Affiliations:** 1 Department of Neurology, Copenhagen University Hospital Bispebjerg, Nielsine Nielsensvej 6A & B, 2400 Copenhagen, Denmark; 2 Copenhagen Trial Unit, Centre for Clinical Intervention Research, The Capital Region, Copenhagen University Hospital Rigshospitalet, Copenhagen, Denmark; 3 Department of Neurology, Heidelberg University Hospital, Heidelberg, Germany; 4 Department of Regional Health Research, The Faculty of Heath Sciences, University of Southern Denmark, Odense, Denmark; 5 Department of Cardiology, Holbæk Hospital, Holbæk, Denmark; 6 Department of Neurology, Klinikum Frankfurt Höchst, Frankfurt, Germany

**Keywords:** Prothrombin complex concentrate, Anticoagulants, Vitamin K antagonist, Direct oral anticoagulants, Bleeding, Systematic review

## Abstract

**Background:**

Swift reversal of oral anticoagulation is deemed essential for the outcome of patients with anticoagulation-related critical bleeding. The aim of this systematic review was to evaluate the benefits and harms of prothrombin complex concentrate (PCC) in patients with oral anticoagulants-related critical bleeding.

**Methods:**

For this systematic review CENTRAL, MEDLINE, Embase, LILACS, BIOSIS, Web of Science, and clinical trial registries were systematically searched. Clinical study reports were also requested from competent authorities. Eligible for inclusion were randomised clinical trials comparing PCC versus no intervention, placebo, or other reversal interventions in participants with critical bleeding related to ongoing treatment with vitamin K antagonist (VKA) or direct oral anticoagulants (DOAC). Pre-specified primary outcomes were all-cause mortality, health-related quality of life, and serious adverse events for which meta-analyses, Trial Sequential Analysis, and GRADE assessments were conducted.

**Results:**

Three trials, randomising a total of 291 participants, evaluated PCC against two different active comparators in participants with VKA-related critical bleeding, and two trials, randomising a total of 534 participants, evaluated PCC against two different active comparators in participants with factor Xa-related critical bleeding. Among participants with VKA-related critical bleeding, meta-analyses showed no evidence of a difference between PCC versus fresh frozen plasma (FFP) when assessing all-cause mortality (risk ratio [RR] 1.05; 95% confidence interval (CI) 0.27 to 4.05; low certainty), health-related quality of life (mean difference 1.04; 95% CI − 0.94 to 3.02; very low certainty), and serious adverse events (RR 1.33; 95% CI 0.94 to 1.88; very low certainty), but information is currently sparse. Among participants with factor Xa-related critical bleeding, PCC could not be shown superior or inferior to other reversal strategies (FFP or andexanet alfa) on any patient-relevant outcome, but information is currently sparse.

**Conclusion:**

Among participants with VKA or DOAC-related critical bleeding, evidence from randomised clinical trials is currently insufficient to establish if PCC is superior or inferior versus other interventions in decreasing the risk of undesirable patient-relevant outcomes or improving health-related quality of life.

**Supplementary Information:**

The online version contains supplementary material available at 10.1186/s13049-025-01334-1.

## Introduction

The use of oral anticoagulation treatment is increasing [1–3]. A major concern when administering oral anticoagulants to patients is the risk of critical bleeding and especially the risk of intracranial haemorrhage. Until 2008, the only option for oral anticoagulation treatment was vitamin K antagonists (VKA) [4]. During the last two decades, direct oral anticoagulants (DOACs) have replaced VKA as first-line therapy within several indications, e.g. non-valvular atrial fibrillation [1, 3, 5]. Even if DOACs are used increasingly, the use of the older drug-class of VKA is not expected to be phased out within the foreseeable future due to a number of unique indications (e.g. prosthetic heart valves) necessitating its use [3–6].

Randomised clinical trials have indicated that the incidence of critical bleeding events is generally lower among patients taking DOAC compared with VKA [7, 8], but the absolute risk of critical bleeding in individuals treated with DOAC is still far from neglectable with an estimated event rate of 2 to 4 events per 100 person years [9–13]. As the number of patients being prescribed anticoagulation treatment increases [1–4], clinically beneficial methods for swift reversal of anticoagulation treatment seem essential for patient safety.

Prothrombin complex concentrate (PCC) is a concentrate of coagulation factors II, IX, and X (3-factor PCC) or II, VII, IX, and X (4-factor PCC) [14]. The concentration of coagulation factors in PCC is approximately 25 times greater than in human plasma [14]. In addition, activated PCC containing enhanced levels of activated coagulation factors has been developed [15]. Guidelines from European and American medical societies [16–19] recommend using PCC to reverse the effect of VKA based on data from randomised clinical trials [20–22]. Guidelines [16–19, 23, 24] and expert opinions [25, 26] also recommend PCC to reverse the anticoagulating effect of DOAC, if specific antidotes cannot be procured. We conducted an extensive systematic review of randomised clinical trials assessing the effect of PCC versus placebo, no treatment, or other treatment strategies in patients with critical bleeding events while undergoing treatment with VKA or DOAC.

## Methods

This systematic review was conducted and reported in accordance with the Preferred Reporting Items for Systematic Reviews and Meta-analysis guidelines (PRISMA) [27] and the Cochrane Handbook of Systematic Reviews of Interventions [28]. Prior to the systematic literature search, this review was registered at the International Prospective Register for Systematic Reviews (PROSPERO) (CRD42018084371), and the review protocol was peer-reviewed and published [29].

### Study selection

Eligible for inclusion were randomised clinical trials comparing PCC versus placebo, no interventions, or other reversal interventions in participants suffering from critical bleeding while undergoing treatment with oral anticoagulants. Critical bleeding was defined as internal or external haemorrhage indicating acute reversal of the coagulopathy inflicted by the oral anticoagulant. Oral anticoagulants were defined as vitamin K antagonists (Anatomical Therapeutic Chemical [ATC] classification B01AA), direct oral anticoagulating agents (direct thrombin inhibitors [ATC classification B01AE], or factor Xa inhibitors [ATC classification B01AF]).

### Data sources and search

A comprehensive literature search was conducted of the online information databases MEDLINE, Embase, Cochrane Central Register of Controlled Trials (CENTRAL), Science Citation Index (Web of Science), Latin American and Caribbean Literature on Health Sciences (LILACS), and BIOSIS from inception to May 2024. We also searched the trial registries ClinicalTrials.gov, World Health Organisation International Clinical Trials Registry Platform (ICTRP), European Union Clinical Trials Register, International Standard Randomised Controlled Trial Number (ISRCTN) Registry, Australian New Zealand Clinical Trials Register (ANZCTR), Clinical Trials Register—India, National Institute of Public Health Clinical Trials Search (Japan), and Chinese Clinical Trial Registry (ChiCTR). The complete search strategies are presented in additional file 1.

To search for unpublished clinical trials and additional information on published clinical trials, national and multinational competent authorities were applied for access to clinical study reports supplied by pharmaceutical companies during application for marketing authorisations. We requested the competent authorities to release all clinical study reports on any type of prothrombin complex concentrate versus placebo, no interventions, or other interventions in participants taking any kind of oral anticoagulants supplied to the competent authorities from the year 2000 to the time of application. Competent authorities in all individual member states in the European Union, the European Medicines Agency as well as national competent authorities in the United States of America, Canada, Norway, Iceland, United Kingdom, Liechtenstein, China, India, Japan, Australia, and New Zealand were contacted (see additional file 2).

Two authors independently screened records from online information databases and documents received from competent authorities for eligible randomised clinical trials (JG and CO). Discrepancies were solved by discussion or mediated by a third author (HC). Trial registries were searched by a single author (CO). In addition to the search strategy, reference lists of identified publications were checked as well as related systematic reviews for additional trials that might be relevant for the present review. No restrictions on language or publication status were imposed. Authors of unpublished relevant trials were contacted for information and offered to supply any available data.

### Data collection and outcome measures

The following primary outcomes were prespecified in the published systematic review protocol [29]: all-cause mortality, health-related quality of life (any continuous outcome scale used by trialists), and proportion of participants with ≥ 1 serious adverse event (defined by International Conference on Harmonisation Guideline for Good Clinical Practice 1997). All primary outcomes were evaluated at longest follow-up. Prespecified secondary outcomes: poor functional outcome (any valid dichotomised scale used by trialist), thromboembolic events, allergic reaction, and pulmonary oedema. All secondary outcomes were evaluated at longest follow-up. Finally, a number of exploratory outcomes were defined: tardy international normalised ratio (INR) correction defined as participants not achieving reversal to a predefined INR cut point within 3 h after infusion start (if data from the 3-h cut point were not available, reported INR correction between 0.5 and 6 h after infusion start could be included), poor clinical haemostatic efficacy (prolonged ongoing bleeding or haematoma expansion) as defined by trialists (the assessment closest to 24 h after admission was used if multiple assessments were reported), and proportion of participants receiving ≥ 1 transfusion with packed red blood cells (during longest follow-up). The chosen exploratory outcomes were assigned as exploratory, as they had no direct patient-relevance. All outcome analyses were evaluated separately among participants with VKA-related critical bleeding and DOAC-related critical bleeding. The outcome ‘tardy INR correction’ was only evaluated among participants with VKA-related critical bleeding.

Participants with intracranial haemorrhage were compared to participants with all other types of bleeding locations in subgroup analysis. Other prespecified subgroup analyses were not possible due to a paucity of published data.

Two authors (JP and CO) independently extracted data from the identified randomised clinical trials and assessed the risk of bias. The risk of bias assessments were performed in accordance with the Cochrane risk-of-bias tool (RoB 1) [28, 30] and Lundh et al. [31] (Table 1 and additional file 5). Trials were assessed as high risk of vested interest bias in case of any industry funding including unrestricted grants. Besides the overall risk of bias for each trial, we also assessed the bias-domains’Blinding of outcome assessors’,’Incomplete outcome data’, and’Selective outcome reporting’ for each outcome individually (see additional file 6). The two authors resolved differences by discussion or by involving a third author (JCJ). The corresponding authors of the identified trials were contacted by email in case of missing data, missing protocol, or unclear/ambiguous information. The interventions from the identified trials were reported in accordance with The Template of Intervention Description and Replication (TIDieR) [32] (see additional file 4).

**Table 1:** Characteristics of included randomised clinical trials

Trial	Boulis et al	Sarode et al	Steiner et al	Shadvar et al	Connolly et al
Year	1999	2013	2016	2021	2024
Trial characteristics					
Number of trial sites	1	36	5	ND	131
Current anticoagulation treatment on admission					
*VKA*	21	216	54	0	0
*DOAC*	0	0	0	41	530*
Biochemical criteria for inclusion	PT > 17 s at the time of randomisation	INR ≥ 2.0 within 3 h before study treatment	Admission INR ≥ 2.0	None	None
Population	Intracranial haemorrhage	Major bleeding	Spontaneous intracerebral or subdural haemorrhage	Major bleeding	Intracranial haemorrhage
Intervention					
*PCC*	4-factor, not activated, PCC (Konyne, Bayer, Elkhart, Indiana, USA)	4-factor, not activated, PCC (Beriplex P/N, CSL Behring, Marburg, Germany)	4-factor, not activated, PCC (Octaplex, Octapharma, Lachen, Switzerland)	4-factor, not activated, PCC (Octaplex, Octapharma, Canada)	At the discretion of the investigator
*Control*	No intervention	Fresh frozen plasma	Fresh frozen plasma	Fresh frozen plasma	Andexanet alfa
Co-interventions	10 mg subcutaneous vitamin K and fresh frozen plasma at the maximum tolerated dose	5 to 10 mg intravenous vitamin K (or according to local guidelines)	10 mg intravenous vitamin K	None	None
Rescue intervention	None	None	PCC administration in both intervention arms if INR > 1.2 at 3 h after first intervention	An additional dose of PCC in those allocated to PCC or an additional dose of fresh frozen plasma in those allocated to fresh frozen plasma	At the discretion of the investigator
Protocol or prospective registration available	No	Yes	Yes	Yes	Yes
Allocation in trial					
Allocated to PCC (no. of participants)	8	107	28	20	230
Allocated to control (no. of participants)	13	109	26	21	263
Risk of bias assessment†					
Allocation sequence generation	Unclear risk	Low risk	Low risk	Unclear risk	Low risk
Allocation concealment	Unclear risk	Low risk	Low risk	Unclear risk	Low risk
Blinding of participants and treatment providers	High risk	High risk	High risk	Unclear risk	High risk
Blinding of outcome assessors	High risk	Low risk	Low risk	Unclear risk	Low risk
Incomplete outcome data	High risk	Low risk	Low risk	Unclear risk	High risk
Selective outcome reporting	Unclear risk	Unclear risk	Low risk	Unclear risk	Low risk
Vested interest bias	Unclear risk	High risk	High risk	Unclear risk	High risk

Through description of the utilised interventions and control treatments in each trial is presented in supplementary analysis as per the TIDieR recommendations

*VKA* Vitamin K Antagonists, *INR* International Normalised Ratio, *PCC* Prothrombin complex concentrate, *ND* Not disclosed

^*^Only participants receiving either prothrombin complex concentrate or andexanet alfa were formally eligible for this review

^†^Detailed risk of bias appraisal can be found in additional file 5

### Meta-analysis

Meta-analyses were conducted when data from at least two trials were available. The applied statistical methodology was based on the recommendations in the Cochrane Handbook of Systematic Review of Interventions [28] and the eight-step assessment proposed by Jakobsen and colleagues [33]. Relative risks were calculated for dichotomous outcomes and mean difference for continuous outcomes (both with 95% confidence intervals). Effect estimates from individual trials were combined, using both fixed-effect and random-effects models (most conservative estimate chosen as primary result). Random-effects meta-analysis were performed using the DerSimonian and Laird approach [34]. Heterogeneity of the effect estimates was assessed by visual inspection of the forest plots and by the inconsistency (I^2^) statistic. The influence of attrition and incomplete outcome data were assessed by ‘best–worst case’ and ‘worst-best case’ scenarios. In these analyses, it was alternatingly assumed that those with missing outcome in the PCC group had either suffered/not-suffered the outcome, and those in the control group had not-suffered/suffered the outcome (see additional file 7). Beta-binominal regression was used as a supplementary analysis, if trials reported zero-events (see additional file 8) [35]. All meta-analyses were carried out in Stata 18 (StataCorp, TX, USA).

### Control of random errors in meta-analysis

To control for inflation of the familywise type 1 error due to testing of multiple outcomes (3 primary and 4 secondary), the *α*-level (type 1 error risk) was adjusted to 0.0125 (0.05/4) as previously recommended [33]. If more than one trial provided evidence for an outcome, we used Trial Sequential Analysis to control the risk of random error. Within the framework of the Trial Sequential Analysis (Copenhagen Trial Unit, 0.9.5.10 Beta) [36], we calculated a diversity-adjusted required information size (DARIS). In calculating DARIS, we assumed a type 1 error rate of 1.25%, a type 2 error rate of 10%, as well as a quantification of diversity (heterogeneity) (D^2^) based on the present meta-analysis of the outcome. For all dichotomous outcomes, a minimally relevant effect equal to a relative risk reduction of 20% was pragmatically assumed and an incidence equal to the incidence observed in the control arm of the meta-analysis of the outcome. For continuous outcomes, a minimally relevant effect equal to the standard deviation (SD) divided by two was assumed. Only when the total number of included participants surpasses the DARIS, PCC can in meta-analysis be declared either superior to the control intervention based on an *α*-level of 1.25% or equivalent based on a *β*-level of 10%. When the total number of included participants does not reach the DARIS, the *α*-level is penalised using Lan-DeMets’ implementation of the O’Brian-Flemming *α*-spending function [36]. This penalisation maintains the approximate overall desired type 1 error rate, as the trials are added sequentially to the meta-analysis [37]. Before the DARIS is reached, PCC can only be declared superior compared with control, if the significance level exceeds the penalised *α*-level for benefit [33].

Bayes factor indicates the ratio between the likelihood of the observed data conditional on the null-hypothesis and the likelihood of the observed data conditional on the assumed minimally relevant effect [33, 38]. It consequently measures the ratio between the statistical evidence for the null-hypothesis and the statistical evidence for the minimally relevant effect. Bayes factor less than 0.1 (10 times more likely under the minimally relevant effect) was used as a threshold for significance [33].

### Certainty of evidence

Grading of Recommendation, Assessment, Development and Evaluation (GRADE) approach were utilised to assess the certainty of the conclusions associated with each of the seven patient-important primary and secondary outcomes [39, 40]. It was prespecified that if no subgroup differences were detected when comparing pooled effect estimates between trials at low (or if no, at relatively lower) risk of bias to trials at high risk of bias, the summary of findings tables would be based on the overall analysis.

## Results

### Included trials

**Fig. 1:**
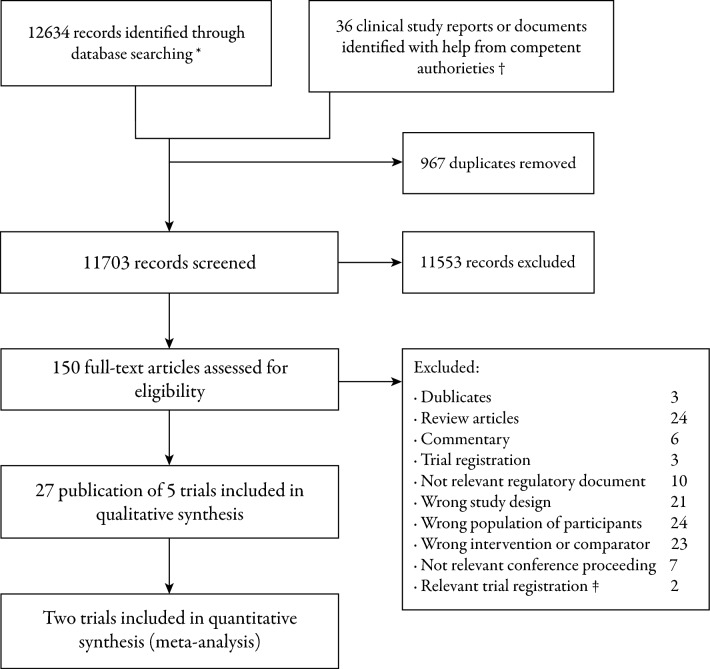
PRISMA flow diagram. Flow of information through the systematic review. *Detailed search strategy is listed in supplementary material. †The full process of applying competent authorities for clinical study reports is presented in supplementary material. ‡ The details of the trials are available in additional file 3

Through our literature search, 12,670 records were identified (Fig. 1). After screening and full-text review, 27 publications detailing five randomised clinical trials were included. The five trials randomised a total of 825 participants eligible for this systematic review. Three trials evaluated PCC against two different active comparators in participants with VKA-related critical bleeding, and two trials evaluated PCC against two different active comparators in participants with factor Xa-related critical bleeding. No identified trials evaluated PCC against no treatment or placebo in participants with anticoagulation-related critical bleedings. Four additional trials might contain potential eligible (subgroups of) participants. The trialists/sponsors were contacted (no data provided [see additional file 3]).

The characteristics of the included trials are presented in Table 1. All trials were parallel group, open-label trials. All trials were deemed at high risk of bias (Table 1 and additional file 5). The exact definition of the outcomes and length of follow-up used in the individual trials are presented in additional file 6.

### PCC versus fresh frozen plasma (FFP) in VKA-related critical bleeding

**Fig. 2:**
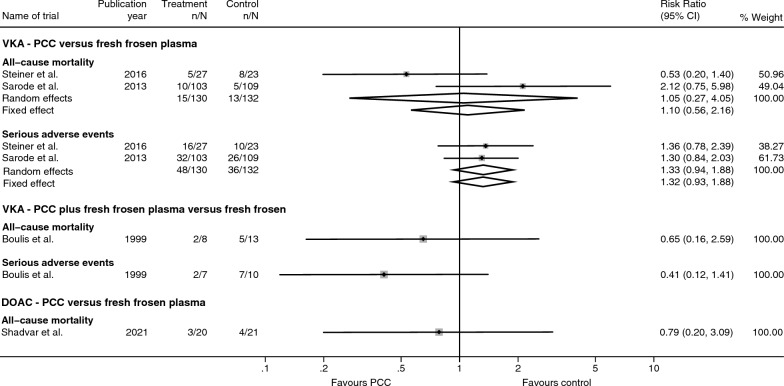
Dichotomous primary outcomes. Forest-plot displaying the results from meta-analyses of primary dichotomous outcomes. Fixed and random effects estimates displayed. RR—relative risk, CI—confidence intervals, PCC—prothrombin complex concentrate

Two trials randomised participants with VKA-related critical bleeding to PCC versus FFP [21, 22]. Both trials reported on the incidence of all-cause mortality. Random-effects meta-analysis showed no evidence of a difference between PCC and FFP when assessing all-cause mortality (RR 1.05; 95% CI 0.27 to 4.05; *p* = 0.95; Bayes factor [BF] = 1.08; Fig. 2). Heterogeneity was substantial (I^2^ = 72.4%). The risk of outcome-specific bias due to blinding of outcome assessor was generally unclear, the risk of selective outcome reporting was low, and the risk of bias due to incomplete outcome data was low (see additional file 6).

One trial reported on health-related quality of life. No evidence was observed of a difference between PCC and FFP (Mean difference 1.04; 95% CI − 0.94 to 3.02; *p* = 0.30; BF = 0.65). The risk of outcome-specific bias due to blinding of outcome assessor was high, the risk of selective outcome reporting was low (see additional file 6).

Both trials reported on the incidence of serious adverse events. Random-effects meta-analysis showed no evidence of a difference between PCC and FFP when assessing the incidence of serious adverse events (RR 1.33; 95% CI 0.94 to 1.88; *p* = 0.11; BF = 16.4; Fig. 2). No heterogeneity was identified (I^2^ = 0%). The risk of outcome-specific bias due to blinding of outcome assessor was unclear, the risk of selective outcome reporting was low, and the risk of bias due to incomplete outcome data was high (see additional file 6).

**Fig. 3:**
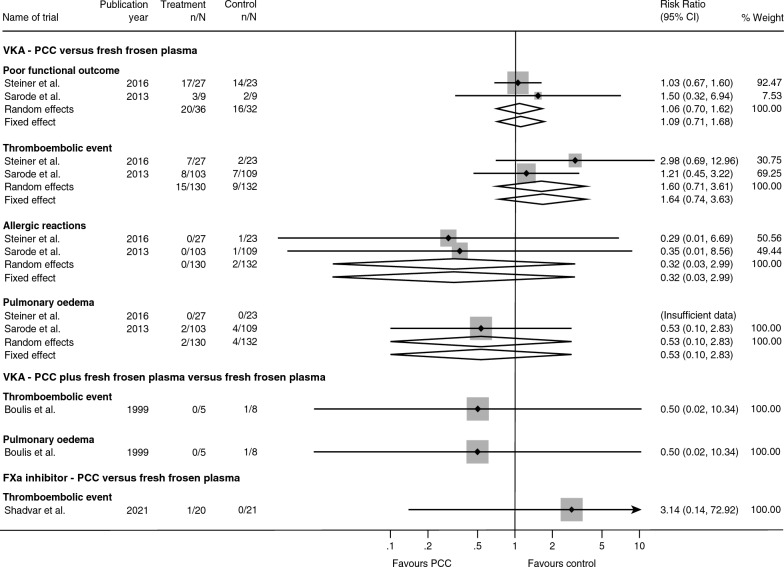
Secondary outcomes. Forest-plot displaying the results from meta-analyses of secondary outcomes. Fixed and random effects estimates displayed. RR—relative risk, CI—confidence intervals, PCC—prothrombin complex concentrate

Both trials reported on poor functional outcome, but only among participants with intracranial haemorrhage. Random-effects meta-analysis showed no evidence of a difference between PCC and FFP when assessing the risk of poor functional outcome (RR 1.06; 95% CI 0.70 to 1.62; *p* = 0.77; BF = 2.32; Fig. 3). The risk of outcome-specific bias due to blinding of outcome assessors was unclear, the risk of selective outcome reporting was low, and the risk of bias due to incomplete outcome data was estimated to be low (see additional file 6).

Both trials reported on the incidence of thromboembolic events, allergic reactions, and pulmonary oedema [21, 22] (Fig. 3). Random-effects meta-analysis showed no evidence of a difference between PCC versus FFP in the risk of thromboembolic events (RR 1.60; 95% CI 0.71 to 3.61; *p* = 0.26; BF = 2.11), allergic reactions (RR 0.32; 95% CI 0.03 to 2.99 *p* = 0.32; BF = 0.84), or pulmonary oedema (RR 0.53; 95% CI 0.10 to 2.83; *p* = 0.46; BF = 0.85). Risk of bias for lack of blinding was unclear, and the risk of selective outcome reporting was low for all outcomes. Risk of bias due to incomplete outcome data was estimated to be high for thromboembolic events and low for allergic reactions and pulmonary oedema (see additional file 6).

In Trial Sequential Analysis, the acquired information size was not large enough to confirm or reject that administration of PCC (versus FFP) is associated with a 20% relative risk reduction in any of the primary or secondary outcomes presented above (see additional file 9). Certainty of evidence was assessed as very low or low for all outcomes (see additional file 11).

**Fig. 4:**
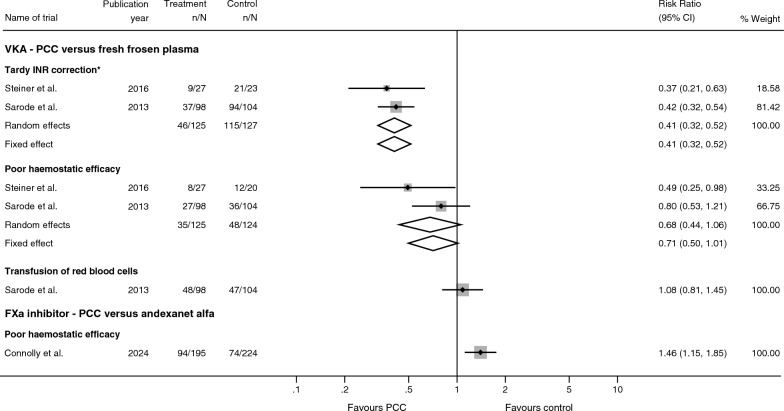
Exploratory outcomes. Forest-plot displaying the results from meta-analyses of exploratory outcomes. Fixed and random effects estimates displayed. Full definition and timing of outcome assessments in each trial can be found in additional file 6. RR—relative risk, CI—confidence intervals, PCC—prothrombin complex concentrate. * Steiner et al. measured INR-levels 3 h after start of infusion and Sarode et al. measured INR-levels 0.5 h after end of infusion

Both trials reported on tardy INR correction. Meta-analysis showed strong evidence that PCC is superior to FFP in limiting the incidence of tardy INR correction (RR 0.41; 95% CI 0.32 to 0.52; *p* < 0.001; BF < 0.001, Fig. 4). No statistical or visual heterogeneity was apparent (I^2^ = 0%). Trial Sequential Analysis showed that the DARIS was not reached, but that the Z-curve crossed the superiority boundary (see additional file 9). Risk of bias due to lack of blinding of outcome assessor was estimated to be low, risk of selective outcome reporting was low and risk of bias due to incomplete outcome data was low (see additional file 6).

Both trials reported on clinical haemostatic efficacy [21, 22]. Random-effects meta-analysis showed no formal statistical evidence of a difference between PCC versus FFP in preventing poor clinical haemostatic efficacy (RR 0.68; 95% CI 0.44 to 1.06; *p* = 0.09; BF = 0.30). Heterogeneity was moderate (I^2^ = 27.0%). In Trial Sequential Analysis, the current information sizes were not large enough to confirm or reject that administration of PCC was associated with a 20% relative risk reduction in the incidence of poor clinical haemostatic efficacy (see additional file 9). Risk of bias due to blinding of outcome assessor was low, risk of selective outcome reporting was low, and risk of bias due to incomplete outcome data is estimated to be high (see additional file 6).

One trial reported on transfusion of red blood cells [21]. The trial showed no evidence of a difference between PCC versus FFP in the risk of needing transfusion with red blood cells (RR 1.08; 95% CI 0.81 to 1.45; *p* = 0.59; BF = 6.84). Risk of bias due to blinding of outcome assessor was estimated to be unclear, risk of selective outcome reporting was low, and risk of bias due to incomplete outcome data was low (see additional file 6).

For the outcomes all-cause mortality, serious adverse events, thromboembolic events, tardy INR-correction, and poor clinical haemostatic efficacy, the effect of PCC versus FFP was assessed exploratorily between participants with intracranial haemorrhage compared to other bleeding sites (see additional file 10). No significant heterogeneity between subgroups was identified in any of the analyses.

### PCC plus FFP versus FFP alone in VKA-related critical bleeding

One trial evaluated PCC plus FFP versus FFP alone among participants with VKA-related critical bleedings [20]. The trial showed no evidence of a difference between PCC plus FFP versus FFP alone on all-cause mortality (RR 0.65; 95% CI 0.16 to 2.59; *p* = 0.54; BF = 0.87; Fig. 2) or serious adverse events (RR 0.41; 95% CI 0.12 to 1.41; *p* = 0.17; BF = 0.65). The risk of outcome-specific bias due to lack of blinding of outcome assessor was high for both outcomes, and the risk of bias due to incomplete outcome data was low for both outcomes (see additional file 6).

Of the secondary outcomes, only data on thromboembolic events and pulmonary oedema were reported. The trial showed no evidence of a difference between PCC plus FFP versus FFP alone in the risk of thromboembolic events (RR 0.50; 95% CI 0.02 to 10.34; *p* = 0.65; BF = 0.95; Fig. 3) or pulmonary oedema (RR 0.50; 95% CI 0.02 to 10.34; *p* = 0.65; BF = 0.95). The risk of bias due to lack of blinding was high for both outcomes. Risk of bias due to incomplete outcome data was low (see additional file 6). Certainty of evidence for all outcomes were assessed as very low certainty of evidence (see additional file 11).

### PCC versus FFP in factor Xa-related critical bleeding

One trial evaluated PCC versus FFP among participants with factor Xa-related critical bleeding [41]. The trial showed no evidence of a difference between PCC versus FFP in the risk of all-cause mortality (RR 0.79; 95% CI 0.20 to 3.09; *p* = 0.73; BF = 0.94; Fig. 2) or thromboembolic events (RR 3.14; 95% CI 0.14 to 72.92; *p* = 0.48; BF = 1.12). The risk of outcome-specific bias due to lack of blinding of outcome assessor was unclear for both outcomes, and the risk of bias due to incomplete outcome data was low for both outcomes (see additional file 6). Both outcomes were assessed as very low certainty of evidence (see additional file 11).

### PCC versus andexanet alfa in factor Xa-related critical bleeding

One trial evaluated andexanet alfa versus usual care among participants with factor Xa-related critical bleeding [42], but 86% of the usual care group received PCC (PCC dosed according to investigators decision). Only those receiving PCC in the usual care group were formally eligible for this review, and no direct comparison between PCC versus andexanet alfa on any patient relevant outcome was published by the trialists (we report data for usual care [86% of whom received PCC] versus andexanet alfa for primary and secondary outcomes below for completion). The only direct comparison between the participants receiving PCC versus andexanet alfa published by the trialists was clinical haemostatic efficacy.

The trial showed that administration of PCC (versus andexanet alfa) was associated with a higher incidence of poor clinical haemostatic efficacy (RR 1.46; 95% CI 1.15 to 1.85; *p* = 0.002; BF = 1810.8; Fig. 4). Risk of bias due to blinding of outcome assessor was low, and risk of bias due to incomplete outcome data is estimated to be high (see additional file 6).

When comparing usual care (86% of whom received PCC) versus andexanet alfa, the trial showed no evidence of a difference in the risk of all-cause mortality (RR 0.92; 95% CI 0.69 to 1.22; *p* = 0.55; BF = 1.31) or poor function outcome (modified Rankin Scale > 3) (RR 0.96; 95% CI 0.86 to 1.07; *p* = 0.47; BF = 106.5). The trial did, however, show borderline evidence for a lower incidence of thromboembolic events among patients allocated to usual care (86% of whom received PCC) versus andexanet alfa (RR 0.55; 95% CI 0.30 to 1.00; *p* = 0.052; BF = 0.32).

## Discussion

Among participants with VKA-related critical bleeding, no evidence was observed that administration of PCC was associated with a decreased risk of any undesirable patient-relevant outcomes nor an improvement in health-related quality of life. However, large uncertainty is still attached to the conclusions in this review, as the certainty of the evidence is generally low to very low (largely due to imprecision and risks of bias). Among participants with VKA-related critical bleeding, the review conclusively demonstrated that the use of PCC was superior to FFP alone in reversing raised INR, with a statistically insignificant trend towards lower incidence of poor clinical haemostatic efficacy when PCC was used. Among participants with factor Xa-related critical bleeding, no data directly evaluating PCC versus andexanet alfa on patient-relevant outcomes were published by trialists (only data comparing usual care [86% of whom received PCC] with andexanet alfa were published). The data did support that PCC was inferior versus andexanet alfa in preventing poor clinical haemostatic efficacy (preventing haematoma expansion), but also indicate borderline evidence of an increased incidence of thromboembolic events in the andexanet alfa group. No trials evaluating activated PCC against other reversal strategies were identified.

### Strengths and limitations

The strength of this review includes the registration plus publication of the systematic review protocol before the literature search was conducted [29]. A comprehensive search of the literature was performed including a search for unpublished data. This included application to competent authorities for clinical study reports allowing us to obtain information not contained in any of the original trial publications. We employed rigorous inclusion criteria accepting only randomised clinical trials to be able to assess treatment effects in the most unbiased fashion. Our rigorous methodology has made us able to conduct robust assessments of the current evidence for the treatment effect of PCC in the reversal of anticoagulation treatment.

Our review has limitations. The conclusions presented in this review are limited by the relatively small number of participants included in the identified trials. Furthermore, all the trials included in this review were at high risk of bias. As blinding of the trial personnel is generally not feasible when randomising participants to reversal of anticoagulation treatment, all trials used an open label design. As trials with incomplete blinding are at high risk of overestimating the intervention effect (especially when evaluating subjective outcome measures) [43], it is not unlikely that bias might affect the results of this meta-analysis. Due to the paucity of trials, we were not able to perform all the prespecified subgroup analyses. Further, due to the heterogeneity and small sample sizes of trials, it was not possible to include dosing in the analysis.

### Comparison with other studies

Among participants with VKA-related critical bleedings, PCC was conclusively superior compared with FFP in normalising INR, but this did not seem to translate into benefit on patient relevant outcomes. Treatment effects on putative surrogate outcomes should generally not be accepted, before comparable effects are demonstrated on patient-relevant outcomes [44]. It is, however, likely that swift normalisation of INR might be more important within certain patient populations. In patients with intracranial haemorrhage, quick haemostasis is likely paramount to prevent expansion of the intracranial haematoma and more extensive brain damage. Data from observational studies support that fast normalisation of INR might translate into a decreased risk of intracranial haematoma expansion [45, 46], which aligns with this review demonstrating a trend towards better clinical haemostatic efficacy among participants with VKA-related bleedings receiving PCC. Among participants with intracerebral haemorrhage, recent trials have demonstrated that implementation of interventions aiming to prevent haematoma expansion early after symptom onset results in improved functional outcome [47, 48].

It is a well-known fact that patients undergoing reversal of anticoagulation treatment are at a risk of thromboembolic complications [49]. Patients, who are prescribed anticoagulation treatment, will per se be at high risk of thromboembolic events (e.g. due to atrial fibrillation, previous venous thromboembolism, or mechanical heart valves), and discontinuation of the anticoagulation treatment might expose the patient to risk. This thromboembolic risk is likely amplified by administration of pro-haemostatic agents. Based on our review and published high-quality data [49], it remains uncertain if PCC is associated with a higher rate of thromboembolic complication compared with FFP in patients with VKA-related critical bleeding.

Transfusion of plasma can cause well-known transfusion-related adverse events such as transfusion-related acute lung injury (TRALI) or transfusion associated circulatory overload (TACO) [50, 51]. Both will often manifest as pulmonary oedema [50]. Among patients undergoing reversal of VKA therapy using FFP, an observational study has indicated an overall 19% incidence of pulmonary complication (TRALI, TACO, and unspecified pulmonary oedema) [52]. The authors reported the risk of pulmonary complications to increase in a dose-dependent manner [52]. A 19% risk of pulmonary complications appears to be higher than the number observed in this review.

Previous systematic reviews have evaluated the question of PCC for reversal of VKA-related critical bleeding [53–55]. All reviews support the use of PCC over other interventions for rapid INR reduction in patients with critical bleeding while undergoing treatment with VKA [53–55], but some also report superiority of PCC on patient-relevant outcomes (reduced mortality) [54, 55]. In our opinion, these reviews contain methodological shortcomings. Two of the systematic reviews were not prospectively registered [53, 55], and all included unadjusted effect estimates from observational studies [53–55]. Inclusion of observational data (especially with no control of confounding) in a meta-analysis of intervention effects is problematic, as empirical studies have shown that observational studies are prone to provide biased treatment effect estimates due to confounding and methodological biases [56–58]. A Cochrane review updated in 2015 evaluated PCC for reversal of VKA treatment in bleeding and non-bleeding patients [59]. The authors concluded that not enough information was currently present to favour PCC over other reversal strategies [59].

Previous systematic reviews have also included non-bleeding participants needing reversal of anticoagulation treatment due to urgent surgery [54, 59]. We chose to limit our review to trials recruiting participants with critical bleeding. This decision was based on the probability of clinical heterogeneity between patient-populations and between clinical setting (acute setting with bleeding participants compared to non-bleeding participants in need of semi-urgent surgery). Three published trials have evaluated PCC against FFP for the indication of reversal of VKA prior to subacute or acute surgery [60–62]. Two of these trials included only participants needing reversal of VKA treatment prior to cardiac surgery [61, 62]. The trial by Goldstein et al. [60] recruited from a broad spectrum of patients needing reversal of VKA treatment due to urgent surgical or invasive procedures. All three trials recruiting participants in need of urgent surgery support our conclusion that administration of PCC leads to a more rapid INR reversal compared with FFP [60–62]. The trial by Goldstein et al. [60] also found that the risk of thromboembolic events was well balanced between participants allocated to PCC and FFP, however, with a higher probability of pulmonary oedema in participants allocated to FFP. We have corresponded with the sponsors of surgical trials, where at least some of the participants could fit our inclusion criteria (Supplement 4). No data have been provided by the sponsors.

In our systematic review, we identified two randomised clinical trials evaluating PCC against other active comparators in participants with DOAC-related critical bleeding. Both trials recruiting only participants pre-treated with factor Xa-inhibitors [41, 42]. Some observational studies have reported a more favourable prognosis for patients pre-treated with DOAC as compared to VKA [63, 64], whereas others have found a comparable prognosis [65]. Even though some studies indicate a more favourable prognosis of DOAC-related haemorrhage, the mortality is still considerable [63, 65, 66]. PCC for the reversal of DOAC has been evaluated in a number of studies in healthy participants [67] and in animal models [68], which have indicated that the coagulopathy associated with the administration of DOAC can be reversed, at least with reasonable success, using PCC. The mechanism by which PCC is thought to reverse the effect of DOAC is by supplying excessive amounts of the coagulation-factors prothrombin, factor VII, factor IX, and factor X. When the level of thrombin or factor Xa surpasses the inhibition inflicted by the DOAC, a normal coagulation will be reconstituted. Uncontrolled observational data on the haemostatic efficacy and safety of PCC in patients with DOAC-related critical bleeding events indicate that a large proportion of patients achieve good haemostatic effect after receiving PCC [69, 70]. In our present review both identified trials provided no direct evidence that PCC was superior or inferior to other reversal strategies on any patient-relevant outcome, but the trial of Andexanet Alfa in Acute Intracranial Hemorrhage in Patients Receiving an Oral Factor Xa Inhibitor (ANNEXA-I) did report borderline evidence of a higher incidence of thromboembolic events among those allocated andexanet alfa (versus usual care). The finding that andexanet alfa is potentially associated with increased risk of thromboembolism is supported by a meta-analysis combining the results of ANNEXA-I with propensity-score matched studies [71]. In ANNEXA-I, PCC was directly shown to be inferior compared with andexanet alfa in preventing poor clinical haemostatic efficacy, but future trials will need to show if this finding translates into benefit on patient-relevant outcomes or whether this superior haemostatic efficacy is offset by the increased risk of thromboembolic events.

We recommend that future trials randomising participants with either VKA or DOAC-related critical bleeding (especially patients with intracranial haemorrhages) should pay particular attention to limiting the delay from symptom onset to treatment in order to optimise the reversal treatment’s ability to limit haematoma growth. In addition, trials should consider including protocols for co-interventions focusing on early optimal blood-pressure control to facilitate the haemostatic effect of the reversal treatment. Future trials should explore, if early and structured use of mechanical or pharmacological prophylaxis for venous thromboembolism can limit the incidence of thromboembolisms after reversal treatment.

### Comparison with international treatment guidelines

Our systematic review demonstrates a current lack of evidence from randomised clinical trials supporting that administration of PCC is associated with a decreased risk of any undesirable patient-relevant outcomes nor an improvement in health-related quality of life. Furthermore, our systematic review demonstrates that the available randomised clinical trials potentially could be affected by bias. This lack of evidence should be acknowledged and used to motivate future high-quality clinical trials and should not inspire therapeutic nihilism. Several treatment guidelines and consensus documents from scientific associations have evaluated the question of reversal of oral anticoagulation within different categories of critical bleeding patients [16–19, 72]. The guidelines unanimously recommend the use of PCC as first-line treatment in patients with VKA-related life-threatening critical bleeding [16–19, 72]. FFP is reserved as a second-line treatment.

Newer international guidelines generally suggest the use of four-factor PCC or activated PCC (FEIBA®, Baxter International Inc., Deerfield, IL, USA) in DOAC-related critical bleeding, if specific antidotes cannot be procured [16–19, 72, 73]. Currently, two specific antidotes for DOAC are available for clinical use—idarucizumab (Boehringer Ingelheim Pharmaceuticals, Ridgefield, CT, USA; for reversal of dabigatran) and andexanet alfa (AstraZeneca, Cambridge, UK; for reversal of factor Xa inhibitors). As documented in this review, current evidence does not support the superiority of andexanet alfa over PCC on any patient-relevant outcome, but potential safety issues related to administration of andexanet alfa have been flagged [42]. No evidence from randomised clinical trials exists comparing idarucizumab versus PCC. Only a prospective case-series has evaluated idarucizumab [74], demonstrating the ability of the antidote to reverse anticoagulation effect assessed by biochemical coagulation assays and that clinical haemostasis could be achieved in a large proportion of participants. In addition to the efficacy of the antidotes per se*,* other important factors to consider in relation to an effective implementation of the new antidotes in clinical use, are their availability and how fast they can be procured (especially in a rural setting) compared to other more readily reversal agents such as PCC.

## Conclusion

Insufficient evidence from randomised clinical trials is currently available to establishing if treatment with PCC is conclusively superior or inferior to other reversal treatments in improving clinical prognosis or decreasing the risk of serious adverse events among patients with anticoagulation-related critical bleeding. PCC was shown conclusively superior compared with FFP in reversing INR among participants with VKA-related critical bleeding with a trend towards better clinical haemostatic efficacy. PCC was shown to be inferior to andexanet alfa in obtaining clinical haemostatic efficacy in patients with factor Xa-related critical bleedings, but future trials will need to establish, if this effect is offset by a higher incidence of thromboembolic events.

## Supplementary Information

Additional file 1.

Additional file 2.

Additional file 3.

Additional file 4.

Additional file 5.

Additional file 6

Additional file 7.

Additional file 8.

Additional file 9.

Additional file 10.

Additional file 11.

## Data Availability

The dataset on which the results presented in this manuscript are based can be obtained from the corresponding author upon request.

## Abbreviations

PCC: Prothrombin complex concentrate
FFP: Fresh frozen plasma
VKA: Vitamin K antagonist
DOAC: Direct oral anticoagulants
GRADE: Grading of recommendation, assessment, development and evaluation
PRISMA: Preferred reporting items for systematic reviews and meta-analyses
ATC: Anatomical therapeutic chemical (classification system)
RoB: Risk of bias
TIDieR: Template for intervention description and replication
INR: International normalised ratio
