# Supplementary material for: Prothrombin complex concentrate for reversal of oral anticoagulants in patients with oral anticoagulation-related critical bleeding: a systematic review of randomised clinical trials
Source: Scand J Trauma Resusc Emerg Med. 2025 Feb 4;33:19. doi: 10.1186/s13049-025-01334-1 (PMC11792222; doi:10.1186/s13049-025-01334-1)
Supplement: Supplementary file 1 — Additional file 1. [file 13049_2025_1334_MOESM1_ESM.pdf]

Additional file 1:

Supplement 1: Complete literature search strategy

Presented below are the complete search syntaxes used to search online literature databases

Date of search *May 28, 2024*

Cochrane Central Register of Controlled Trials in the Cochrane Library (2024, Issue 4) (1785 hits in CENTRAL)

#1 MeSH descriptor: [Anticoagulants] this term only
#2 (anticoagulant* or OAT* or OAC*):ti,ab,kw
#3 MeSH descriptor: [Vitamin K] this term only
#4 MeSH descriptor: [International Normalized Ratio] this term only
#5 (vitamin* NEAR/3 (antagonist* or k-antagonist*)):ti,ab,kw
#6 VKA*:ti,ab,kw
#7 (dicoumarol* or phenindione* or warfarin* or phenprocoumon* or acenocoumarol* or ethyl biscoumarol or clorindione* or diphenadione* or tioclomarol* or coumarin*):ti,ab,kw
#8 (NOAC* or DOAC* or TSOAC* or ODI* or SODA*):ti,ab,kw
#9 (non-vitamin K oral or new oral or novel oral or direct oral or target-specific oral or oral direct inhibitor or specific oral direct):ti,ab,kw
#10 (factor NEAR/3 (IIa* or Xa*)):ti,ab,kw
#11 (thrombin NEAR/3 inhibitor*):ti,ab,kw
#12 (rivaroxaban* or apixaban* or edoxaban* or betrixaban* or apixaban* or dabigatran*):ti,ab,kw
#13 #1 or #2 or #3 or #4 or #5 or #6 or #7 or #8 or #9 or #10 or #11 or #12
#14 MeSH descriptor: [Hemostatics] explode all trees
#15 MeSH descriptor: [Blood Coagulation Factors] this term only
#16 MeSH descriptor: [Factor IX] this term only and with qualifier(s): [therapeutic use - TU]
#17 MeSH descriptor: [Prothrombin] this term only and with qualifier(s): [therapeutic use - TU]
#18 Blood Coagulation Factor:ti,ab,kw
#19 Prothrombin complex concentrate:ti,ab,kw
#20 (Prothrombin* NEAR/6 (concentrate* or complex*)):ti,ab,kw
#21 (PCC* or 4F-PCC* or 3F-PCC* or aPPC* or Kaskadil or Beriplex or Cofact or Confidex or Kcentra or Ocplex or Octaplex or konyne or PPSB* or Profilnine* or ProthoRAAS or Prothrom* or Proplex* or Pushu Laishi or TachoSil or Uman*):ti,ab,kw
#22 ((Activated* or anti-inhibitor*) NEAR/6 (prothrombin or complex or concentrate)):ti,ab,kw
#23 (Factor VIII Inhibitor Bypassing Activity or FEIBA or Factor IX complex concentrate or FIXCC*):ti,ab,kw
#24 #14 or #15 #19 or #20 or #21 or #22 or #23
#25 #13 and #24

MEDLINE Ovid (1946 to 28 May 2024) (2623 hits)

1. Anticoagulants/
2. (anticoagulant$ or OAT$ or OAC$).ti,ab.
3. Vitamin K/ai [Antagonists & Inhibitors]
4. International Normalized Ratio/
5. (vitamin$ adj3 (antagonist$ or k-antagonist$)).ti,ab.
6. VKA$.ti,ab.
7. (dicoumarol$ or phenindione$ or warfarin$ or phenprocoumon$ or acenocoumarol$ or ethyl biscoumarol or clorindione$ or diphenadione$ or tioclomarol$ or coumarin$).ti,ab.
8. (NOAC$ or DOAC$ or TSOAC$ or ODI$ or SODA$).ti,ab.
9. (non-vitamin K oral or new oral or novel oral or direct oral or target-specific oral or oral direct inhibitor or specific oral direct).ti,ab.
10. (factor adj3 (IIa$ or Xa$)).ti,ab.
11. (thrombin adj3 inhibitor$).ti,ab.
12. (rivaroxaban$ or apixaban$ or edoxaban$ or betrixaban$ or $xaban$ or dabigatran$).ti,ab.
13. 1 or 2 or 3 or 4 or 5 or 6 or 7 or 8 or 9 or 10 or 11 or 12
14. exp Hemostatics/

15. Blood Coagulation Factors/
16. Factor IX/tu [Therapeutic Use]
17. Prothrombin/tu [Therapeutic Use]
18. Blood Coagulation Factor.ti,ab.
19. Prothrombin complex concentrate.ti,ab.
20. (Prothrombin$ adj6 (concentrate$ or complex$)).ti,ab.
21. (PCC$ or 4F-PCC$ or 3F-PCC$ or aPPC$ or Kaskadil or Beriplex or Cofact or Confidex or Kcentra or Ocplex or Octaplex or konyne or PPSB$ or Profilnine$ or ProthoRAAS or Prothrom$ or Proplex$ or Pushu Laishi or TachoSil or Uman$).ti,ab.
22. ((Activated$ or anti-inhibitor$) adj6 (prothrombin or complex or concentrate)).ti,ab.
23. ('Factor VIII Inhibitor Bypassing Activity' or FEIBA or Factor IX complex concentrate or FIXCC$).ti,ab.
24. 14 or 15 or 16 or 17 or 18 or 19 or 20 or 21 or 22 or 23
25. 13 and 24
26. (random* or blind* or placebo* or meta-analys*).mp. [mp=title, abstract, original title, name of substance word, subject heading word, floating sub-heading word, keyword heading word, organism supplementary concept word, protocol supplementary concept word, rare disease supplementary concept word, unique identifier, synonyms]
27. 25 and 26

Embase Ovid (1974 to 28 May 2024) (8054 hits)
1. anticoagulant agent/
2. (anticoagulant$ or OAT$ or OAC$).ti,ab.
3. exp coumarin/
4. (vitamin$ adj3 (antagonist$ or k-antagonist$)).ti,ab.
5. VKA$.ti,ab.
6. (dicoumarol$ or phenindione$ or warfarin$ or phenprocoumon$ or acenocoumarol$ or ethyl biscoumarol or clorindione$ or diphenadione$ or tioclomarol$ or coumarin$).ti,ab.
7. (NOAC$ or DOAC$ or TSOAC$ or ODI$ or SODA$).ti,ab.
8. (non-vitamin K oral or new oral or novel oral or direct oral or target-specific oral or oral direct inhibitor or specific oral direct).ti,ab.
9. exp blood clotting factor 10a inhibitor/
10. exp thrombin inhibitor/
11. (factor adj3 (IIa$ or Xa$)).ti,ab.
12. (thrombin adj3 inhibitor$).ti,ab.
13. (rivaroxaban$ or apixaban$ or edoxaban$ or betrixaban$ or $xaban$ or dabigatran$).ti,ab.
14. 1 or 2 or 3 or 4 or 5 or 6 or 7 or 8 or 9 or 10 or 11 or 12 or 13
15. exp hemostatic agent/
16. Blood Coagulation Factor.ti,ab.
17. Prothrombin complex concentrate.ti,ab.
18. (Prothrombin$ adj6 (concentrate$ or complex$)).ti,ab.
19. (PCC$ or 4F-PCC$ or 3F-PCC$ or aPPC$ or Kaskadil or Beriplex or Cofact or Confidex or Kcentra or Ocplex or Octaplex or konyne or PPSB$ or Profilnine$ or ProthoRAAS or Prothrom$ or Proplex$ or Pushu Laishi or TachoSil or Uman$).ti,ab.
20. ((Activated$ or anti-inhibitor$) adj6 (prothrombin or complex or concentrate)).ti,ab.
21. (Factor VIII Inhibitor Bypassing Activity or FEIBA or Factor IX complex concentrate or FIXCC$).ti,ab.
22. 15 or 16 or 17 or 18 or 19 or 20 or 21
23. 14 and 22
24. (random* or blind* or placebo* or meta-analys*).mp. [mp=title, abstract, heading word, drug trade name, original title, device manufacturer, drug manufacturer, device trade name, keyword, floating subheading word, candidate term word]
25. 23 and 24
26. limit 25 to human

LILACS (Bireme) (1982 to 28 May 2024) (380 hits)
((anticoagulant$ or OAT$ or OAC$) or (vitamin$ and (antagonist$ or k-antagonist$)) or VKA$ or (dicoumarol$ or phenindione$ or warfarin$ or phenprocoumon$ or acenocoumarol$ or ethyl biscoumarol or clorindione$ or diphenadione$ or tioclomarol$ or coumarin$) or (NOAC$ or DOAC$ or TSOAC$ or ODI$ or SODA$) or (non-vitamin K oral or new oral or novel oral or direct oral or target-specific oral or oral direct inhibitor or specific oral direct) or (factor and (IIa$ or Xa)) or (thrombin and inhibitor$) or (rivaroxaban$ or apixaban$ or edoxaban$ or betrixaban$ or apixaban$ or dabigatran$)) [Words] and (Blood Coagulation Factor or Prothrombin complex concentrate or (Prothrombin$ and (concentrate$ or complex$)) or (PCC$ or 4F-PCC$ or 3F-PCC$ or aPPC$ or Kaskadil or Beriplex or Cofact or Confidex or Kcentra or Ocplex or Octaplex or konyne or PPSB$ or Profilnine$ or ProthoRAAS or Prothrom$

or Proplex$ or Pushu Laishi or TachoSil or Uman$) or ((Activated$ or anti-inhibitor$) and (prothrombin or complex or concentrate)) or (Factor VIII Inhibitor Bypassing Activity or FEIBA or Factor IX complex concentrate or FIXCC$)) [Words]

BIOSIS (Web of Science) (1969 to 28 May 2024) (1208 hits)
#5 #4 AND #3
#4 TS=(random* or blind* or placebo* or meta-analys*)
#3 #2 AND #1
#2 TS=(Blood Coagulation Factor or Prothrombin complex concentrate or (Prothrombin* NEAR/6 (concentrate* or complex*)) or (PCC* or 4F-PCC* or 3F-PCC* or aPPC* or Kaskadil or Beriplex or Cofact or Confidex or Kcentra or Ocplex or Octaplex or konyne or PPSB* or Profilnine* or ProthoRAAS or Prothrom* or Proplex* or Pushu Laishi or TachoSil or Uman*) or ((Activated* or anti-inhibitor*) NEAR/6 (prothrombin or complex or concentrate)) or (Factor VIII Inhibitor Bypassing Activity or FEIBA or Factor IX complex concentrate or FIXCC*))
#1 TS=((anticoagulant* or OAT* or OAC*) or (vitamin* NEAR/3 (antagonist* or k-antagonist*)) or VKA* or (dicoumarol* or phenindione* or warfarin* or phenprocoumon* or acenocoumarol* or ethyl biscoumarol or clorindione* or diphenadione* or tioclomarol* or coumarin*) or (NOAC* or DOAC* or TSOAC* or ODI* or SODA*) or (non-vitamin K oral or new oral or novel oral or direct oral or target-specific oral or oral direct inhibitor or specific oral direct) or (factor NEAR/3 (IIa* or Xa)) or (thrombin NEAR/3 inhibitor*) or (rivaroxaban* or apixaban* or edoxaban* or betrixaban* or apixaban* or dabigatran*))

Science Citation Index Expanded (1900 to 28 May 2024) and Conference Proceedings Citation Index – Science (1990 to 28 May 2024) (Web of Science) (1494 hits)
#5 #4 AND #3
#4 TS=(random* or blind* or placebo* or meta-analys*)
#3 #2 AND #1
#2 TS=(Blood Coagulation Factor or Prothrombin complex concentrate or (Prothrombin* NEAR/6 (concentrate* or complex*)) or (PCC* or 4F-PCC* or 3F-PCC* or aPPC* or Kaskadil or Beriplex or Cofact or Confidex or Kcentra or Ocplex or Octaplex or konyne or PPSB* or Profilnine* or ProthoRAAS or Prothrom* or Proplex* or Pushu Laishi or TachoSil or Uman*) or ((Activated* or anti-inhibitor*) NEAR/6 (prothrombin or complex or concentrate)) or (Factor VIII Inhibitor Bypassing Activity or FEIBA or Factor IX complex concentrate or FIXCC*))
#1 TS=((anticoagulant* or OAT* or OAC*) or (vitamin* NEAR/3 (antagonist* or k-antagonist*)) or VKA* or (dicoumarol* or phenindione* or warfarin* or phenprocoumon* or acenocoumarol* or ethyl biscoumarol or clorindione* or diphenadione* or tioclomarol* or coumarin*) or (NOAC* or DOAC* or TSOAC* or ODI* or SODA*) or (non-vitamin K oral or new oral or novel oral or direct oral or target-specific oral or oral direct inhibitor or specific oral direct) or (factor NEAR/3 (IIa* or Xa)) or (thrombin NEAR/3 inhibitor*) or (rivaroxaban* or apixaban* or edoxaban* or betrixaban* or apixaban* or dabigatran*))

Supplement 2: Complete trial registries search strategy

Clinicaltrials.gov (www.clinicaltrials.gov) – searched 14. July 2024

Search terms	Number of hits
Prothrombin	2017
Factor IX	400
PCC OR Kaskadil OR Beriplex OR Cofact OR Confidex OR Kcentra OR Ocplex OR Octaplex OR konyne OR PPSB OR Profilnine OR ProthoRAAS OR Prothrom OR Proplex OR Pushu Laishi OR Uman OR FEIBA	501

WHO ICTRP (http://apps.who.int/trialsearch/) – searched 14. July 2024

Search terms	Number of hits
Prothrombin	147
Factor IX	174
PCC OR Kaskadil OR Beriplex OR Cofact OR Confidex OR Kcentra OR Ocplex OR Octaplex OR konyne OR PPSB OR Profilnine OR ProthoRAAS OR Prothrom OR Proplex OR Pushu Laishi OR Uman OR FEIBA	164

Clinicaltrialsregister.eu (www.clinicaltrialsregister.eu) – searched 14. July 2024

Search terms	Number of hits
Prothrombin	576
Factor IX	107
PCC OR Kaskadil OR Beriplex OR Cofact OR Confidex OR Kcentra OR Ocplex OR Octaplex OR konyne OR PPSB OR Profilnine OR ProthoRAAS OR Prothrom OR Proplex OR Pushu Laishi OR TachoSil OR Uman OR FEIBA	82

ANZCTR (www.anzctr.org.au) – searched 14. July 2024

Search terms	Number of hits
Prothrombin	320
Factor IX	41
PCC OR Kaskadil OR Beriplex OR Cofact OR Confidex OR Kcentra OR Ocplex OR Octaplex	35
konyne OR PPSB OR Profilnine OR ProthoRAAS OR Prothrom OR Proplex OR Pushu Laishi OR TachoSil	1
Uman OR FEIBA	2

Clinical Trials Registry – India (http://ctri.icmr.org.in/) – searched 14. July 2024

Search terms	Number of hits
Prothrombin	6
Factor IX	5

NIPH (https://rctportal.niph.go.jp)– searched 14. July 2024

Search terms	Number of hits
Prothrombin	164

Factor IX	8
FEIBA	0
PCC	299

ISRCTN (https://www.isrctn.com)– searched 14. July 2024

Search terms	Number of hits
Prothrombin	160
Factor IX	9
PCC OR Kaskadil OR Beriplex OR Cofact OR Confidex OR Kcentra OR Ocplex OR Octaplex OR konyne OR PPSB OR Profilnine OR ProthoRAAS OR Prothrom OR Proplex OR Pushu Laishi OR TachoSil OR Uman OR FEIBA	38<br><br>(terms searched individually)

ChiCTR (http://www.chictr.org.cn) – searched 30. July 2024

Search terms	Number of hits
Prothrombin	5
Factor IX	1
PCC OR Kaskadil OR Beriplex OR Cofact OR Confidex OR Kcentra OR Ocplex OR Octaplex OR konyne OR PPSB OR Profilnine OR ProthoRAAS OR Prothrom OR Proplex OR Pushu Laishi OR TachoSil OR FEIBA	7<br><br>(terms searched individually)
