# Supplementary material for: Prothrombin complex concentrate for reversal of oral anticoagulants in patients with oral anticoagulation-related critical bleeding: a systematic review of randomised clinical trials
Source: Scand J Trauma Resusc Emerg Med. 2025 Feb 4;33:19. doi: 10.1186/s13049-025-01334-1 (PMC11792222; doi:10.1186/s13049-025-01334-1)
Supplement: Supplementary file 2 — Additional file 2. [file 13049_2025_1334_MOESM2_ESM.pdf]

Additional file 2:

Supplement 3: Search for clinical study reports with national and multinational competent authorities.

**Country**	**Name of authority**	**Initial contact**	**Response**
**North America**			
United States of America	U.S Food and Drug Administration	Initially contacted on 28.1.2022 via Freedom of Information Act request form (https://www.accessdata.fda.gov/scripts/foi/FOIRequest/requestform.cfm)	Reply received on 1.2.2022. FDA supplied three publicly available documents.<br><br>FDA informs that the application is being triaged to the complex queue of the Centre for Biologics Evaluation and Research. The expected waiting time before the request will come up for processing is 18-24 months.
Canada	Health Canada	Initially contacted on 23.9.2024 via email (hcinfo.infosc@canada.ca).	Reply received on 7.11.2024.<br><br>Health Canada informs that only Canadian citizens, permanent residents, individuals who are present in Canada, and corporations present in Canada can file a access-to-information request.
**Europe**			
European Union	European Medicines Agency	Initially contacted on 23.1.2022 via website application (https://www.ema.europa.eu/en/about-us/contact/send-question-european-medicines-agency).	Reply received on 2.2.2022.<br><br>European Medicines Agency replies that only one prothrombin complex concentrate product (Nonafact, Sanquin Plasma Products, Amsterdam, Netherlands) has been centrally authorised. According to the European public assessment report [1], Nonafact has been studied in two clinical studies recruiting participants with haemophilia B (not within the scope of this review).
Austria	Austrian Agency for Health and Food Safety	Initially contacted on 7.9.2021 via email (anfragen@ages.at).	Reply received on 4.2.2022.<br><br>The Austrian competent authority replies that the Austrian legislation does not entail a freedom of information act. The Austrian competent authority, consequently, denies disclosing clinical study reports.<br><br>Beriplex (CSL Behring, King of Prussia, PA, USA), Cofact (Sanquin Plasma Products, Amsterdam, Netherlands), Octaplex (Octapharma, Lachen, Switzerland), FEIBA (Baxter Healthcare Corp, IL, USA) and Prothromplex TOTAL (Baxalta Innovations, Vienna, Austria) are all registered in Austria via decentralised procedure (mutual recognition) [2-6]. Either Sweden, Denmark, or Croatia is also concerned member state on all mutual recognition procedures. It is therefore concluded that all documents of interest are likely obtained from other national competent authorities (SE, HR, DK)<br><br>Prothromplex Partiell registered nationally but with same marketing authorisation holder and manufacturer as Prothromplex TOTAL (Takeda Manufacturing Austria AG).

Country	Name of authority	Initial contact	Response
Belgium	Federal Agency for Medicines and Health Products	Initially contacted on 7.9.2021 via email (welcome@fagg-afmps.be).	No reply to initial contact received.<br><br>Reminder sent on 20.1.2022 to same email address. No response received.<br><br>Cofact (Sanquin Plasma Products, Amsterdam, Netherlands), Confidex (CSL Behring, King of Prussia, PA, USA), Octaplex (Octapharma, Lachen, Switzerland) and Prothromplex TOTAL (Baxalta Innovations, Vienna, Austria) are all registered in Belgium via decentralised procedure (mutual recognition) [3-5, 7]. Either Sweden, Denmark, or Croatia are also concerned member state on all mutual recognition procedures. It is therefore concluded that all documents of interest are already obtained from. It is therefore concluded that all documents of interest are likely obtained from other national competent authorities (SE, HR, DK).
Bulgaria	Bulgarian Drug Agency	Initially contacted on 7.9.2021 via email (bda@bda.bg).	No reply to initial contact received.<br><br>Reminder sent on 20.1.2022 to same email address. No response received.<br><br>Octaplex (Octapharma, Lachen, Switzerland), Beriplex (CSL Behring GmbH), FEIBA (Baxter Healthcare Corp, IL, USA) and Prothromplex TOTAL (Baxalta Innovations, Vienna, Austria) are all registered in Bulgaria via decentralised procedure (mutual recognition) [3, 5-7]. Either Sweden, Denmark, or Croatia is also concerned member state on all mutual recognition procedures. It is therefore concluded that all documents of interest are likely obtained from other national competent authorities (SE, HR, DK). Advanced search on registered products in Bulgaria not possible.
Croatia	Agency for Medicinal Products and Medical Devices	Initially contacted on 7.9.2021 via email (halmed@halmed.hr).	Reply received on 15.9.2021.<br><br>The Croatian competent authority replies that the following products currently is authorised for marketing in Croatia.<br>1. Beriplex (CSL Behring, King of Prussia, PA, USA)<br>2. Octaplex (Octapharma, Lachen, Switzerland)<br>3. FEIBA (Baxalta Innovations, Vienna, Austria)<br><br>The Croatian competent authority also stresses that Beriplex (CSL Behring, King of Prussia, PA, USA) and FEIBA (Baxalta Innovations, Vienna, Austria) have been approved through decentralised procedures in the European Union (mutual recognition) with Croatia as concerned member state [8, 9] (Sweden also concerned member state). As a result we applied for all documents related to Octaplex (Octapharma, Lachen, Switzerland) and FEIBA (Baxalta Innovations, Vienna, Austria).<br><br>The Croatian competent authority releases 8 documents on Octaplex (Octapharma, Lachen, Switzerland) and FEIBA (Baxalta Innovations, Vienna, Austria).
Cyprus	Ministry of Health - Pharmaceutical Services	Initially contacted on 7.9.2021 via email (phscentral@phs.moh.gov.cy).	No reply to initial contact received.

Country	Name of authority	Initial contact	Response
			Reminder sent on 20.1.2022 to same email address. The Cypriot competent authority replies that no prothrombin complex concentrate products are authorised through national registration procedure. Beriplex (CSL Behring, King of Prussia, PA, USA), FEIBA (Baxter Healthcare Corp, IL, USA) and Octaplex (Octapharma, Lachen, Switzerland) are all approved through decentralised procedures in the European Union (mutual recognition) with Cyprus as concerned member state (Denmark also concerned member state) [5-7].
Czech Republic	State Institute for Drug Control	Initially contacted on 7.9.2021 via email (infs@sukl.cz).	Reply received on 15.9.2021.<br><br>The Czech competent authority replies that it cannot disclose clinical study reports.<br><br>Octaplex (Octapharma, Lachen, Switzerland), Beriplex (CSL Behring, King of Prussia, PA, USA), FEIBA (Baxter Healthcare Corp, IL, USA) and Prothromplex TOTAL (Baxalta Innovations, Vienna, Austria) are all registered in Czech Republic via decentralised procedure (mutual recognition) [3, 5-7]. Either Sweden, Denmark, or Croatia is also concerned member state on all mutual recognition procedures. It is therefore concluded that all documents of interest are likely obtained from other national competent authorities (SE, HR, DK).
Denmark	Danish Medicines Agency	Initially contacted on 19.9.2021 via email (dkma@dkma.dk).	Reply received on 21.9.2021.<br><br>The Danish competent authority has issued marketing authorisation for the following products.<br>1. Confidex (CSL Behring, King of Prussia, PA, USA)<br>2. Octaplex (Octapharma, Lachen, Switzerland)<br>3. Prothromplex TOTAL (Baxalta Innovations, Vienna, Austria)<br><br>As these products are all approved through decentralised procedures in the European Union [2, 3, 10, 11] (mutual recognition) with Denmark as a concerned member state the Danish competent authority is supplied with a list of the documents already obtained from other national competent authorities.<br><br>The Danish Medicines Agency releases six documents concerning Confidex (CSL Behring, King of Prussia, PA, USA) and Octaplex (Octapharma, Lachen, Switzerland).
Estonia	Estonia State Agency of Medicines	Initially contacted on 7.9.2021 via email (info@ravimiamet.ee).	Reply received 8.9.2021.<br><br>The Estonian competent authority replies that according to its internal regulation it cannot disclose clinical study reports.<br><br>Octaplex (Octapharma, Lachen, Switzerland), FEIBA (Baxter Healthcare Corp, IL, USA) and Prothromplex TOTAL (Baxalta Innovations, Vienna, Austria) are all registered in Estonia via decentralised procedure (mutual recognition) [3, 5]. Either Sweden or Denmark is also concerned member state on all mutual recognition procedures. It is therefore concluded that all documents of interest are likely obtained from other national competent authorities (SE, HR, DK).

Country	Name of authority	Initial contact	Response
Finland	Finnish Medicines Agency	Initially contacted on 7.9.2021 via email (Esa.Heinonen@fimea.fi).	Reply received on 5.10.2021.<br><br>The Finnish competent authority replies that the following products currently has a marketing authorisation in Finland:<br>1. Cofact (Sanquin Plasma Products, Amsterdam, Netherlands)<br>2. Confidex (CSL Behring, King of Prussia, PA, USA)<br>3. Octaplex and Octanine (Octapharma, Lachen, Switzerland)<br>4. Prothromplex TOTAL (Baxalta Innovations, Vienna, Austria) (discontinued due to sunset regulation).<br>5. Bemofil (Finnish Red Cross Blood Service, Helsinki, Finland)<br><br>Finnish competent authority advice that we apply for the clinical study reports directly from the holders of the marketing authorisations. In case the Finnish Medicines Agency would decide to disclose the material, the agency would most likely charge 'multiple thousands of euros' to do so. The request was not pursued any further.<br><br>According to the Heads of Medicines Agencies and the European Medicines Agency Confidex (CSL Behring, King of Prussia, PA, USA), Cofact (Sanquin Plasma Products, Amsterdam, Netherlands) and Octaplex (Octapharma, Lachen, Switzerland) are all approved through decentralised procedures in the European Union [2, 4, 10] (mutual recognition) with Finland as concerned member state (Denmark also concerned member state on Confidex and Octaplex, Sweden concerned member state on Cofact).
France	National Agency for the Safety of Medicine and Health Products	Initially contacted on 7.9.2021 via email (dajr@ansm.sante.fr).	No reply to initial contact received.<br><br>Reminder sent on 20.1.2022 to same email address. No response received.<br><br>Cofact (Sanquin Plasma Products, Amsterdam, Netherlands), Confidex (CSL Behring, King of Prussia, PA, USA), and Octaplex (Octapharma, Lachen, Switzerland) are all registered in France via decentralised procedure (mutual recognition) [4, 5, 7]. Either Sweden, Denmark, or Croatia is also concerned member state on all mutual recognition procedures. It is therefore concluded that all documents of interest are likely obtained from other national competent authorities (SE, HR, DK).<br><br>Kanokad and Kaskadil (LFB-Biomedicaments) has been national registered in 2008 and 1999, respectively.
Germany	Federal Instutute for Drugs and Medical Devices	Initially contacted on 6.9.2021 via website application (https://www.bfarm.de/SiteGlobals/Forms/Kontakt/EN/Kontakt_Seite_2_Formular.html).	Reply received on 10.9.2021.<br><br>The German competent authority replies that the requested documents are available through EudraCT and PharmNet.Bund.<br><br>Octaplex (Octapharma, Lachen, Switzerland), Beriplex (CSL Behring, King of Prussia, PA, USA), FEIBA (Baxter Healthcare Corp, IL, USA) and Prothromplex TOTAL (Baxalta Innovations, Vienna, Austria) are all registered in Germany via decentralised procedure

Country	Name of authority	Initial contact	Response
			(mutual recognition) [3, 5-7]. Either Sweden, Denmark, or Croatia is also concerned member state on all mutual recognition procedures. It is therefore concluded that all documents of interest are likely obtained from other national competent authorities (SE, HR, DK).
Germany	Paul Ehrlich Institute	Initially contacted on 7.9.2021 via email (pei@pei.de).	No reply to initial contact received.<br><br>Reminder sent on 20.1.2022 to same email address. No response received.<br><br>Marketing Applications with coagulation factors are assessed by Paul Ehrlich Institute (PEI), but Paul Ehrlich Institute and Federal Institute for Drugs and Medical Devices have a common register of approved products. Therefore, please refer to the information above.
Greece	National Organization for Medicines	Initially contacted on 7.9.2021 via email (relation@eof.gr).	No reply to initial contact received.<br><br>Reminder sent on 20.1.2022 to same email address. No response received.<br><br>Beriplex (CSL Behring, King of Prussia, PA, USA), FEIBA (Baxter Healthcare Corp, IL, USA) and Prothromplex TOTAL (Baxalta Innovations, Vienna, Austria) are all registered in Greece via decentralised procedure (mutual recognition) [3, 6, 7]. Either Sweden, Denmark, or Croatia is also concerned member state on all mutual recognition procedures. It is therefore concluded that all documents of interest are likely obtained from other national competent authorities (SE, HR, DK).
Hungary	National Institute of Pharmacy and Nutrition	Initially contacted on 30.8.2021 via email (ogyei@ogyei.gov.hu).	Reply received on 17.2.2022.<br><br>The Hungarian competent authority replies that it will not be able to provide new information as all products granted marketing authorisation in Hungary were from multinational manufacturers and, consequently, will be duplicates of material available from other competent authorities. Beriplex (CSL Behring, King of Prussia, PA, USA), Prothromplex TOTAL (Baxalta Innovations, Vienna, Austria) and Octaplex (Octapharma, Lachen, Switzerland) are all approved through decentralised procedures European Union [3, 7, 10] (mutual recognition) with Hungary as concerned member state. Either Sweden, Denmark, or Croatia is also concerned member state on all mutual recognition procedures. It is therefore concluded that all documents of interest are likely obtained from other national competent authorities (SE, HR, DK).
Iceland	Icelandic Medicines Agency	Initially contacted on 30.8.2021 via email (ima@ima.is).	No reply to initial contact received.<br><br>Reminder sent on 20.1.2022 to same email address. No response received.<br><br>Octaplex (Octapharma, Lachen, Switzerland) and Cofact (Sanquin Plasma Products, Amsterdam, Netherlands) are both registered in Iceland via decentralised procedure (mutual recognition) [4, 5]. Either Sweden or Denmark is also concerned member state on all mutual recognition procedures. It is therefore concluded that all documents of interest are likely obtained from other national competent authorities (SE, DK).

Country	Name of authority	Initial contact	Response
Ireland	Health Products Regulatory Authority (HPRA)	Initially contacted on 30.8.2021 via email (info@hpra.ie).	Response received on 1.11.2021. HPRA advice that the request should be submitted as a freedom of information request.<br><br>Freedom of information request submitted 31.1.2022. Reply received on 9.2.22 informing that the FOI is rejected due to the request being to voluminous. The HPRA recommends to resubmit the request specifying International Non-proprietary Names (INN) for the substances of interest. We were unable to find the INN for prothrombin complex concentrate even after consultation with a pharmaceutical regulatory affairs specialist. Request was not pursued any further.<br><br>According to the European Medicines Agency and the Heads of Medicines Agency, Ireland is concerned member state for decentralised procedures [3, 7, 10, 11] (mutual recognition) of three prothrombin complex concentrate products - Beriplex (CSL Behring, King of Prussia, PA, USA), Prothromplex TOTAL (Baxalta Innovations, Vienna, Austria), and Octaplex (Octapharma, Lachen, Switzerland). Either Sweden, Denmark, or Croatia is also concerned member state on all mutual recognition procedures. It is therefore concluded that all documents of interest are likely obtained from other national competent authorities (SE, HR, DK).
Italy	Italian Medicines Agency	Initially contacted on 28.8.2021 via email (ufficiostampaaifa@aifa.gov.it).	No reply to initial contact received.<br><br>Reminder sent on 20.1.2022 to same email address. No response received.<br><br>Cofact (Sanquin Plasma Products, Amsterdam, Netherlands), Confidex (CSL Behring, King of Prussia, PA, USA), Pronativ (Octapharma, Lachen, Switzerland) and Proplex (Baxalta Innovations, Vienna, Austria) are all registered in Italy via decentralised procedure (mutual recognition) [3-5, 7, 11]. Either Sweden, Denmark, or Croatia is also concerned member state on all mutual recognition procedures. It is therefore concluded that all documents of interest are likely obtained from other national competent authorities (SE, HR, DK).<br><br>Protromplex TIM 3 (Baxalta Innovations GmbH) and Uman Complex (Kedrion S.p.A.) are approved via national procedure in 1976. Kedcom (Kedrion S.p.A.) are approved via national procedure in 2013.
Latvia	State Agency of Medicines of the Republic of Latvia	Initially contacted on 28.8.2021 via email (info@zva.gov.lv).	No reply to initial contact received.<br><br>Reminder sent on 20.1.2022 to same email address. No response received.<br><br>Octaplex (Octapharma, Lachen, Switzerland), FEIBA (Baxter Healthcare Corp, IL, USA) and Prothromplex TOTAL (Baxalta Innovations, Vienna, Austria) are all registered in Latvia via decentralised procedure (mutual recognition) [3, 5, 6]. Either Sweden or Denmark is also concerned member state on all mutual recognition procedures. It is therefore concluded that all documents of interest are likely obtained from other national competent authorities (SE, DK).

Country	Name of authority	Initial contact	Response
Liechtenstein	Department of Pharmaceuticals, Office of Health	Initially contacted on 28.8.2021 via email (info.ag@llv.li).	Reply received on 31.8.2021.<br><br>The Office of Health replies that the product in question is approved through decentralised authorisation procedure and that Liechtenstein adopts the Austrian marketing authorisation.
Lithuania	State Medicines Control Agency of Lithuania	Initially contacted on 28.8.2021 via email (vvkt@vvkt.lt).	No reply to initial contact received.<br><br>Reminder sent on 20.1.2022 to same email address. Reply received on 25.1.2022. The Lithuanian competent authority replies that they are not in possession of clinical study reports relevant for the review.<br><br>Octaplex (Octapharma, Lachen, Switzerland), FEIBA (Baxter Healthcare Corp, IL, USA) and Prothromplex TOTAL (Baxalta Innovations, Vienna, Austria) are all registered in Lithuania via decentralised procedure (mutual recognition) [3, 6, 10]. Either Sweden or Denmark is also concerned member state on all mutual recognition procedures. It is therefore concluded that all documents of interest are likely obtained from other national competent authorities (SE, DK).
Luxembourg	Ministry of Health	Initially contacted on 15.8.2021 via email (ministere-sante@ms.etat.lu).	No reply to initial contact received.<br><br>Reminder sent on 20.1.2022 to same email address. No response received.<br><br>Cofact (Sanquin Plasma Products, Amsterdam, Netherlands), Confidex (CSL Behring, King of Prussia, PA, USA), Octaplex (Octapharma, Lachen, Switzerland) and Prothromplex TOTAL (Baxalta Innovations, Vienna, Austria) are all registered in Luxembourg via decentralised procedure (mutual recognition) [3, 4, 7, 10]. Either Sweden, Croatia or Denmark is also concerned member state on all mutual recognition procedures. It is therefore concluded that all documents of interest are likely obtained from other national competent authorities (SE, DK). Ministry of Health in Luxembourg does not issue any marketing authorisations unless the same product is already registered in Belgium, France, Germany or Austria.
Malta	Malta Medicines Authority	Initially contacted on 15.8.2021 via email (info.medicinesauthority@gov.mt).	No reply to initial contact received.<br><br>Reminder sent on 21.1.2022 to same email address. No response received.<br><br>Beriplex (CSL Behring, King of Prussia, PA, USA), FEIBA (Baxter Healthcare Corp, IL, USA) and Prothromplex TOTAL (Baxalta Innovations, Vienna, Austria) are all registered in Malta via decentralised procedure (mutual recognition) [3, 6, 7]. Either Sweden or Denmark is also concerned member state on all mutual recognition procedures. It is therefore concluded that all documents of interest are likely obtained from other national competent authorities (SE, DK).
Netherlands	Dutch Health and Youth Care Inspectorate	Initially contacted on 13.8.2021 via email (meldpunt@igj.nl).	Reply received on 17.8.2021.

Country	Name of authority	Initial contact	Response
			The Dutch Health and Youth Care Inspectorate (HYCI) replies that it is not the competent authority in the Netherlands granting marketing authorisations. HYCI refers to Medicines Evaluation Board.
Netherlands	Medicine Evaluation Board	Initially contacted on 13.8.2021 via web application form (https://fd8.formdesk.com/collegeterbeoordelingvangenees/submit-your-question/?get=1&sidn=3cbce29b50bf43318461b7142c5102ce)	No reply to initial contact received.<br><br>Reminder sent on 20.1.2022 via the same web application form. No response received.<br><br>Reply received on 23.5.2022. The Medicine Evaluation Board replies that it is of the opinion that the information we request is publicly available through published public assessment reports.<br><br>Cofact (Sanquin Plasma Products, Amsterdam, Netherlands), Octaplex (Octapharma, Lachen, Switzerland), FEIBA (Baxter Healthcare Corp, IL, USA), Beriplex (CSL Behring, King of Prussia, PA, USA) and Prothromplex TOTAL (Baxalta Innovations, Vienna, Austria) are all registered in Netherlands via decentralised procedure (mutual recognition) [3, 4, 6, 7, 10]. Either Sweden, Croatia or Denmark is also concerned member state on all mutual recognition procedures. It is therefore concluded that all documents of interest are likely obtained from other national competent authorities (SE, DK).
Poland	Office for Registration of Medicinal Products, Medical Devices and Biocidal Products	Initially contacted on 12.8.2021 via email (gp@urpl.gov.pl).	Reply received on 9.9.2021.<br><br>The polish competent authority requested the application in polish within 60 days otherwise the application would not be processed.<br><br>It was decided not to pursue our application to the polish competent authority any further.<br><br>Octaplex (Octapharma, Lachen, Switzerland), Beriplex (CSL Behring, King of Prussia, PA, USA) and Prothromplex TOTAL (Baxalta Innovations, Vienna, Austria) are all registered in Poland via decentralised procedure (mutual recognition) [3, 7, 10]. Either Sweden, Croatia or Denmark is also concerned member state on all mutual recognition procedures. It is therefore concluded that all documents of interest are likely obtained from other national competent authorities (SE, DK).
Portugal	National Authority of Medicines and Health Products	Initially contacted on 12.8.2021 via email (infarmed@infarmed.pt).	No reply to initial contact received.<br><br>Reminder sent on 20.1.2022 to same email address. No response received.<br><br>Octaplex (Octapharma, Lachen, Switzerland), Beriplex (CSL Behring, King of Prussia, PA, USA) and Prothromplex TOTAL (Baxalta Innovations, Vienna, Austria) are all registered in Portugal via decentralised procedure (mutual recognition) [3, 7, 10]. Either Sweden, Croatia or Denmark is also concerned member state on all mutual recognition procedures. It is therefore concluded that all documents of interest are likely obtained from other national competent authorities (SE, HR, DK).

Country	Name of authority	Initial contact	Response
Romania	National Authority of Medicines and Medical Devices	Initially contacted on 11.8.2021 via email (secretariat@anm.ro).	No reply to initial contact received.<br><br>Reminder sent on 20.1.2022 to same email address. No response received.<br><br>Pronativ (Octapharma, Lachen, Switzerland), Beriplex (CSL Behring, King of Prussia, PA, USA), FEIBA (Baxter Healthcare Corp, IL, USA) and Prothromplex TOTAL (Baxalta Innovations, Vienna, Austria) are all registered in Romania via decentralised procedure (mutual recognition) [3, 6, 7, 10]. Either Sweden, Croatia or Denmark is also concerned member state on all mutual recognition procedures. It is therefore concluded that all documents of interest are likely obtained from other national competent authorities (SE, HR, DK).
Slovakia	State Institute for Drug Control	Initially contacted on 11.8.2021 via email (sukl@sukl.sk).	No reply to initial contact received.<br><br>Reminder sent on 20.1.2022 to same email address. No response received.<br><br>Octaplex (Octapharma, Lachen, Switzerland), Beriplex (CSL Behring, King of Prussia, PA, USA), FEIBA (Baxter Healthcare Corp, IL, USA) and Prothromplex TOTAL (Baxalta Innovations, Vienna, Austria) are all registered in Slovakia via decentralised procedure (mutual recognition) [3, 6, 7, 10]. Either Sweden, Croatia or Denmark is also concerned member state on all mutual recognition procedures. It is therefore concluded that all documents of interest are likely obtained from other national competent authorities (SE, HR, DK).
Slovenia	Agency for Medicinal Products and Medical Devices of the Republic of Slovenia	Initially contacted on 11.8.2021 via email (info@jazmp.si).	No reply to initial contact received.<br><br>Reminder sent on 20.1.2022 to same email address. No response received.<br><br>Octaplex (Octapharma, Lachen, Switzerland), Beriplex (CSL Behring, King of Prussia, PA, USA), FEIBA (Baxter Healthcare Corp, IL, USA) and Proplex (Baxalta Innovations, Vienna, Austria) are all registered in Slovenia via decentralised procedure (mutual recognition) [3, 6, 7, 10]. Either Sweden, Croatia or Denmark is also concerned member state on all mutual recognition procedures. It is therefore concluded that all documents of interest are likely obtained from other national competent authorities (SE, HR, DK).
Spain	Spanish Agency for Medicines and Medical Devices	Initially contacted on 11.8.2021 via email (atencionciudadano@aemps.es).	No reply to initial contact received.<br><br>Reminder sent on 20.1.2022 to same email address. No response received.<br><br>Octaplex (Octapharma, Lachen, Switzerland), Beriplex (CSL Behring, King of Prussia, PA, USA), FEIBA (Baxter Healthcare Corp, IL, USA) and Prothromplex TOTAL (Baxalta Innovations, Vienna, Austria) are all registered in Spain via decentralised procedure (mutual recognition) [3, 6, 7, 10]. Either Sweden, Croatia or Denmark is also concerned member state on all mutual recognition procedures. It is therefore concluded that all documents of interest are likely obtained from other national competent authorities (SE, HR, DK).

Country	Name of authority	Initial contact	Response
Sweden	Swedish Medical Products Agency	Initially contacted on 10.8.2021 via email (registrator@mpa.se).	Reply received on 13.8.2021.<br><br>The Swedish competent authority releases 7 documents concerning:<br>1. Beriplex/Confidex (CSL Behring, King of Prussia, PA, USA)<br>2. Octaplex and Octanine (Octapharma, Lachen, Switzerland)<br>3. Prothromplex TOTAL (Baxalta Innovations, Vienna, Austria) (discontinued due to sunset regulation).<br>4. FEIBA NF (Baxter Healthcare Corp, IL, USA)
United Kingdom	Medicines and Healthcare Products Regulatory Agency	Initially contracted on 18.2.2022 via email (mhracustomerservices@mhra.gov.uk) with freedom of information request.	Reply received on 25.2.2022.<br><br>MHRA rejects the freedom of information request based on the perceived costs related to answering our request. MHRA recommends returning one product licence number we which to see documents for.<br><br>Not pursued any further as documents for the European Medicines Agency indicate that Beriplex (CSL Behring, King of Prussia, PA, USA), Prothromplex TOTAL (Baxalta Innovations, Vienna, Austria) and Octaplex (Octapharma, Lachen, Switzerland) before the United Kingdom's exit from the European Union was granted marketing authorisation through decentralised procedures [12] (mutual recognition) with the United Kingdom as concerned member state. Documents already obtained from Denmark and Sweden. It was deemed unlikely that new information was supplied to the competent authority of the United Kingdom not available from the previously mentioned sources.
**Asia**			
China	National Medical Products Administration	Initially contacted on 29.4.2022 by physical letter as no email address is provided.<br><br>Letter sent to:<br>National Medical Products Administration<br>No 1 Beiluyuan Zhanlan Road<br>Xicheng District<br>Beijing (postal code 100037), China	No reply received.
India	Central Drugs Standard Control Organization	Initially contacted on 28.1.2022 via email (dci@nic.in).	No reply received.
Japan	Pharmaceuticals and Medical Devices Agency (PMDA)	Initially contacted on 29.1.2022 via website application (https://www.pmda.go.jp/cgi-bin/e-contact/contact1.cgi).	Reply received on 7.2.2022.<br><br>The Japanese competent authority (PMDA) replies that the name of the specific documents requested, and their content needs to be specified in a written disclosure request. The PMDA refers to a webpage describing the procedure, fees, and format of the disclosure request only available in Japanese. The PMDA advise that when requesting disclosure of information held by administrative agencies in Japan from countries other than Japan, they strongly advise to delegate such procedures to a proxy in Japan.<br><br>Due to how resource demanding such a request would be it was not pursued.

Country	Name of authority	Initial contact	Response
**Oceania**			
Australia	Therapeutic Goods Administration	Initially contacted on 25.1.2022 via email (tga.foi@health.gov.au).	Reply received on 4.2.2022.<br><br>The Australian competent authority replies that it suggests limiting our scope to one product listed on the Australian Register of Therapeutic Goods. In response to this it was decided to limit our request to Beriplex (CSL Behring, King of Prussia, PA, USA).<br><br>Therapeutic Goods Administration releases 12 documents related to Beriplex (CSL Behring, King of Prussia, PA, USA).
New Zealand	New Zealand Medicines and Medical Devices Safety Authority	Initially contacted on 2.1.2023 via email (medsafeapplications@health.govt.nz).	No reply received.<br><br>Reminder sent on 13.2.2023 to same email address.

Supplementary references

1. European Medicines Agency. Summary of the European public assessment report (EPAR) for Nonafact [Available from: https://www.ema.europa.eu/en/documents/overview/nonafact-epar-summary-public_en.pdf.
2. Heads of Medicines Agencies. Beriplex P/N 250 [Available from: https://mri.cts-mrp.eu/portal/details?productnumber=DE/H/0477/001.
3. Heads of Medicines Agencies. Prothromplex TOTAL 600 I.E. [Available from: https://mri.cts-mrp.eu/portal/details?productnumber=AT/H/0373/001.
4. Heads of Medicines Agencies. Cofact [Available from: https://mri.cts-mrp.eu/portal/details?productnumber=NL/H/0859/001.
5. Heads of Medicines Agencies. Octaplex 500 [Available from: https://mri.cts-mrp.eu/portal/fulltext-search?term=DE%2FH%2F0464%2F001-002.
6. Heads of Medicines Agencies. Feiba 1000 E. [Available from: https://mri.cts-mrp.eu/portal/fulltext-search?term=DE%2FH%2F0464%2F001-002.
7. Heads of Medicines Agencies. Beriplex P/N 500 [Available from: https://mri.cts-mrp.eu/portal/details?productnumber=DE/H/0477/002.
8. Heads of Medicines Agencies. Feiba 500 E. [Available from: https://mri.cts-mrp.eu/portal/details?productnumber=AT/H/0343/001.
9. Heads of Medicines Agencies. Beriplex P/N 1000 [Available from: https://mri.cts-mrp.eu/portal/details?productnumber=DE/H/0477/003.
10. Heads of Medicines Agencies. Octaplex 1000 [Available from: https://mri.cts-mrp.eu/portal/details?productnumber=DE/H/0464/002.
11. European Medicines Agency. List of nationally authorised medicinal products (human prothrombin complex) - PSUSA/00001638/202104 2022 [Available from: https://www.ema.europa.eu/en/documents/psusa/human-prothrombin-complex-list-nationally-authorised-medicinal-products-psusa/00001638/202104_en.pdf.
12. European Medicines Agency. List of nationally authorised medicinal products (human prothrombin complex) - PSUSA/00001638/201604 2017 [Available from: https://www.ema.europa.eu/en/documents/psusa/human-prothrombin-complex-list-nationally-authorised-medicinal-products-psusa/00001638/201604_en.pdf.
