# Supplementary material for: Prothrombin complex concentrate for reversal of oral anticoagulants in patients with oral anticoagulation-related critical bleeding: a systematic review of randomised clinical trials
Source: Scand J Trauma Resusc Emerg Med. 2025 Feb 4;33:19. doi: 10.1186/s13049-025-01334-1 (PMC11792222; doi:10.1186/s13049-025-01334-1)
Supplement: Supplementary file 3 — Additional file 3. [file 13049_2025_1334_MOESM3_ESM.pdf]

Additional file 3:

Supplement 4: Trials with populations or subpopulation potentially fitting the inclusion criteria of this review awaiting assessments

Identifier	Title	Comments
NCT00618098<br><br>www.clinicaltrials.gov (accessed December 16, 2023)	Study of Octaplex (Human Prothrombin Complex Concentrate) and Fresh Frozen Plasma in Patients Under Vitamin K Therapy Antagonist Needing Urgent Surgery or Invasive Procedures	Completed in August 2012. Trial currently unpublished. Data published on clinicaltrials.gov<br><br>Sponsor – Octaphama AG (Lachen, Switzerland) - contacted in August 2018 in order to ascertain if any of the participants fit the inclusion criteria for this review.<br><br>No data provided by the trialists by September 2024.
NCT00803101<br><br>www.clinicaltrials.gov	Four-factor prothrombin complex concentrate versus plasma for rapid vitamin K antagonist reversal in patients needing urgent surgical or invasive interventions: a phase 3b, open-label, non-inferiority, randomised trial. Lancet 2015; 385: 2077 to 2087	Published trial.<br><br>Sponsor - CSL Behring LLC (King of Prussia, PA, USA) – contacted in November 2018, and invited to supply individual or aggregate data on any participants fitting our inclusion criteria.<br><br>No data provided by the trialists by September 2024.
NCT02429453<br><br>www.clinicaltrials.gov (accessed December 16, 2023)	FFP versus PCC in Intracranial hemorrhage.	The trial is listed as withdrawn without any participants enrolled. We attempted to contact the trialists to confirm, however, no reply has been received.
NCT02777424<br><br>www.clinicaltrials.gov (accessed December 16, 2023)	CPP Versus PFC to Correct Coagulation Disorders in Adult Neurosurgical Patients (CLOT-CRANE)	The trial is listed as completed in July 2020. Trial currently unpublished.<br><br>We attempted to contact the trialists, however, no reply has been received.
