# Supplementary material for: Prothrombin complex concentrate for reversal of oral anticoagulants in patients with oral anticoagulation-related critical bleeding: a systematic review of randomised clinical trials
Source: Scand J Trauma Resusc Emerg Med. 2025 Feb 4;33:19. doi: 10.1186/s13049-025-01334-1 (PMC11792222; doi:10.1186/s13049-025-01334-1)
Supplement: Supplementary file 4 — Additional file 4. [file 13049_2025_1334_MOESM4_ESM.pdf]

# Additional file 4:

## Supplement 5: Description of the anticoagulation-reversal intervention in included randomised clinical trials

| Author | Brief Name | Recipient | Why | What (materials and methods) | Who provided | How | Where | When and how much | Tailoring | Modification of Intervention throughout the Trial | Fidelity |
|---|---|---|---|---|---|---|---|---|---|---|---|
| **Steiner et al. (2016)**<br><br>Information based on the main trial publication [1], published trial protocol [2] as well as original trial protocol. | PCC versus FFP in patients with intracranial haemorrhage related to VKA. | Patients admitted with CT-verified intracranial haemorrhage within 12 hours of onset and with an INR of ≥2.0. | Patients with VKA-related intracranial haemorrhage are at particular risk of undergoing haematoma expansion. VKA cause a depletion of functional coagulation factors, and substitution of coagulation factors by administering either FFP or PCC might normalize the coagulation. | Patients were randomized to either FFP (manufactured locally or commercially purchased) or 4-factor, non-activated PCC (Octaplex, Octapharma, Switzerland).<br><br>**FFP arm:** Before administration, participants were blood type tested or AB group plasma was ordered. The plasma was thawed to 37 degrees Celsius. The total dose was calculated as 20mL/kg body weight. Participants received plasma by intravenous infusion as fast as tolerated.<br><br>**PCC arm**: The total initial dose was calculated as 30IU/kg up to a maximum of 3000IU. PCC was intravenously injected at a rate of 1mL per minute initially followed by a rate of 2-3 mL per minute.<br><br>All participants received 10mg of intravenous vitamin K at a rate of <1mg/minute immediately after the start of infusion of the allocated treatment. | Local investigator, doctors and nursing staff. | By direct contact (face-to-face) after acute admission. | After admission, patients underwent CT-scan. The allocated treatment was started within one hour after CT-scan. | The primary intervention was delivered in a single session. This single session consisted of a three hour period from start of the allocated study treatment until first INR measurement.<br><br>If INR >1.2 three hours after initiation of the study treatment, a rescue intervention was initiated. | If INR >1.2 three hours after initiation of the study treatment, a dose of PCC was initiated as a rescue therapy. If INR at three hours was >1.2 but ≤2.0, an additional PCC-dose of 10 IU/kg was administered. If INR at three hours was >2.0 an additional PCC-dose of 30 IU/kg was administered.<br><br>The rescue therapy was initiated regardless of the participant's initial allocation (PCC or FFP).<br><br>10mg of intravenous vitamin-K was repeated every 24 hours until INR≤1.2. | No changes to the protocol were made throughout the trial. | All treatment needed to be documented within the first 24 hours.<br><br>The investigators kept account of all trial medication stored. In addition, the investigator kept account of all trial medication dispensed.<br><br>The site monitor confirmed accountability of all trial medication used. |

| Author | Brief Name | Recipient | Why | What (materials and methods) | Who provided | How | Where | When and how much | Tailoring | Modification of Intervention throughout the Trial | Fidelity |
|---|---|---|---|---|---|---|---|---|---|---|---|
| **Sarode et al. (2013)**<br><br>Information based on main trial publication [3], clinical study report [4] and NCT00708435 (www.clinicaltrials.gov).<br><br>Protocol requested from sponsor (CSL Behring), but not shared. | PCC versus FFP in patients with critical bleeding related to VKA. | Patients experiencing acute critical bleeding while undergoing treatment with VKA and had an INR≥2.0 measured within 3 hours trial intervention. Acute critical bleeding was defined as either:<br>(1) life-threatening bleeding (according to the treating physician)<br>(2) bleeding associated with a drop in hemoglobin ≥2g/dL<br>(3) bleeding requiring transfusion of blood products. | Patients presenting with acute VKA-related critical bleeding require rapid reversal of the coagulopathy by quick restoration of vitamin K-dependent coagulation factors. Replacement of vitamin K-dependent coagulation factors can be achieved by administration of either PCC or FFP. | Participants were allocated to either FFP or 4-factor, non-activated PCC (Beriplex P/N, CSL Behring, Marburg, Germany) by a minimization process ensuring balance in bleeding type and number of patients allocated to each arm (per site and overall).<br><br>**FFP arm:** Participants received FFP according to the dosing algorithm (see tailoring). FFP was infused intravenously at a rate of 1 unit per 30-minute interval.<br><br>**PCC arm:** The total dose of PCC was administered as a single intravenous dose calculated by an algorithm (see tailoring). The maximum infusion rate was 3 IU per kg per minute.<br><br>All patients received 5-10mg intravenous Vitamin K by slow infusion (alternatively the dose and administration route could be tailored in accordance with local guidelines). | Study staff | By direct patient contact (face-to-face) after acute admission. | Not specified. | The primary intervention was delivered in a single session. | The dose of investigational medicinal products was calculated according to body weight and baseline INR (INR obtained ≤3 hours before start of infusion of the IMP).<br><br>**FFP arm** (mL plasma):<br>Baseline INR 2 to <4: 10mL per kg.<br>Baseline INR 4-6: 12mL per kg.<br>Baseline INR >6: 15mL per kg.<br><br>**PCC arm** (IU of factor IX):<br>Baseline INR 2 to <4: 25 IU per kg.<br>Baseline INR 4-6: 35 IU per kg.<br>Baseline INR >6: 50 IU per kg.<br><br>Maximum dose ≤ 5000IU of factor IX or ≤1500mL plasma.<br><br>No per-protocol rescue therapy was used; however, participants were assessed as having poor haemostatic efficacy, if additional doses of hemostatic agents were required. | It is mentioned in clinical study report (BE1116_3002) that Amendment 3.0 (October 15, 2009) included the following change: *"The dose calculation for Beriplex and plasma was modified; the INR closest to start of infusion (up to 3 hours before start of infusion) had to be used for dose calculation" [4]* and *"Regarding concomitant vitamin K1 injections, it was specified that if vitamin K1 had been administered for the current bleeding event prior to enrollment, an additional dose was not needed unless indicated by local clinical practice or the above guidelines."[4]* | None mentioned. |

| Author | Brief Name | Recipient | Why | What (materials and methods) | Who provided | How | Where | When and how much | Tailoring | Modification of Intervention throughout the Trial | Fidelity |
|---|---|---|---|---|---|---|---|---|---|---|---|
| **Boulis et al. (1999)**<br><br>Information based on the main trial publication [5].<br><br>Protocol requested from corresponding author, but no reply received. | Factor IX complex in VKA-related intracranial haemorrhage. | Patients with CT-verified VKA-related intracranial haemorrhage. Trial candidates were required to have a prothrombin time of >17 seconds at the time of randomization. | Patients presenting with intracranial haemorrhage while undergoing treatment with VKA require urgent reversal of the coagulopathy due to the risk of haematoma enlargement as well as due to the risk of intra- and postoperative haemorrhage. | Participants were randomised to either FFP alone or FFP supplemented with factor IX complex concentrate (Konyne, Bayer, Elkhart, Indiana, USA). The factor IX complex concentrate consisted of factor II, factor VII, factor IX, factor X.<br><br>**FFP arm:** After randomization, single-donor plasma was infused intravenously at the maximal pace tolerated by the participants. Participants had a central venous pressure (CVP) monitor inserted in order to detect volume overload. Furosemid was administered intravenously in order to maintain the central venous pressure below 12mmHg. When the patient reached an INR of 1.3, the pace of FFP administration was decreased to 60mL/hour and tapered off during 36 hours.<br><br>**FFP supplemented with factor IX complex concentrate arm:** Participants received the factor IX complex concentrate as soon as it could be thawed. The infusion rate of factor IX complex concentrate was 100IU/min. The participants randomised to factor IX complex concentrate also received FFP at the maximally tolerated rate. Furosemid was given intravenously to maintain euvolemia by balancing FFP infusion with diuresis.<br><br>All participants received 10mg of vitamin K subcutaneously. | Local investigator, doctors and nursing staff. | By direct patient contact (face-to-face) after admission. | After admission, patients underwent CT-scan. Location not specified. | The primary intervention was delivered in a single session. | The dose of investigational medicinal products was calculated according to body weight and baseline INR (INR obtained ≤3 hours before start of infusion of the IMP).<br><br>**PCC arm** (IU of factor IX):<br>Dose was calculated as follows: IU requested = bodyweight (in kg) x (target factor level – current factor level). Factor levels are expressed as a fraction of normal factor levels. Target factor level was set at 50%.<br><br>The current factor levels of the participant was estimated by the INR at the time of randomization: INR 2-3 equal to 10%, INR 3-4 equal to 5% and INR>4 equal to 1%.<br><br>No per-protocol rescue therapy was described. | No documented changes to the intervention were made throughout the trial. | None mentioned. |

| Author | Brief Name | Recipient | Why | What (materials and methods) | Who provided | How | Where | When and how much | Tailoring | Modification of Intervention throughout the Trial | Fidelity |
|---|---|---|---|---|---|---|---|---|---|---|---|
| **Connolly et al. (2024)**<br><br>Information based on the main trial publication and trial protocol [6]. | Andexanet for factor Xa inhibitor-associated acute intracerebral hemorrhage | Patients with intracranial haemorrhage receiving a factor Xa inhibitor within 15 hours prior to randomization. Time from symptom onset to baseline scan was required to be <12 hours. Patients were not eligible if intracerebral haematoma volume was >60mL or Glasgow Coma Scale <7. | Patients presenting with acute factor Xa inhibitor-associated bleeding require rapid reversal of the coagulopathy. | Participants were randomized in a 1:1 ratio to either andexanet alfa (AstraZeneca, Cambridge, UK) or usual care.<br><br>**Andexanet alfa arm:** Andexanet was administered as an IV bolus, followed by a continuous infusion. Participants were either given a 'low dose' consisting of a 400mg bolus over 15 minutes followed by a 480mg infusion over 120 minutes; or a 'high dose' consisting of a 800mg bolus over 30 minutes followed by a 960mg infusion over 120 minutes. Whether a 'low dose' or a 'high dose' were administered depended on the timing and dose of the last factor Xa inhibitor administration.<br><br>**Usual care arm:** For participants randomized to the usual care arm 3- or 4-factor PCC was allowed (in addition to other pro-coagulant factor infusions) | Local investigator, doctors and nursing staff. | By direct patient contact (face-to-face) after admission. | Location not specified. | The primary intervention was delivered in a single session. | In case of clinical deterioration within the first 12 hours after randomization investigators were required to persue additional brain imaging.<br><br>**Andexanet alfa arm:** In case of hematoma expansion no re-dosing of andexanet was allowed, but additional administration of blood products, pro-coagulation factor infusion and hemostatic agents were allowed.<br><br>**Usual care arm:** Any additional administration of blood products, pro-coagulation factor infusion and hemostatic agents (except andexanet) were allowed. | No documented changes to the intervention were made throughout the trial. | The investigators kept account of all trial medication stored. In addition, the investigator kept account of all trial medication dispensed.<br><br>The site monitor confirmed accountability of all trial medication used. |

| Author | Brief Name | Recipient | Why | What (materials and methods) | Who provided | How | Where | When and how much | Tailoring | Modification of Intervention throughout the Trial | Fidelity |
|---|---|---|---|---|---|---|---|---|---|---|---|
| **Shadvar et al. (2021)**<br><br>Information based on main trial publication [7], and IRCT20091012002582N20 (https://irct.behdasht.gov.ir/). | PCC versus FFP in patients with major bleeding related to rivaroxaban. | Patients with rivaroxaban related major bleeding. Trial candidates were required to have taken the last dose of rivaroxaban within 24 hours. | Patients presenting with acute rivaroxaban related major bleeding require rapid reversal of the coagulopathy. | Participants were allocated to either FFP or 4-factor, non-activated PCC (Octaplex, Octapharma, Canada).<br><br>**FFP arm:** The total initial dose was calculated as 10-15mL/kg<br><br>**PCC arm:** The total initial dose was calculated as 25-50IU/kg | Not disclosed | By direct patient contact (face-to-face) after admission. | Not disclosed. | Not disclosed. | In case of ongoing bleeding after administration of the first dose, participants in the PCC group were administered an additional dose of 25IU/kg PCC.<br><br>Participants in the FFP group were administered an additional dose of 7mL/kg FFP in case of ongoing bleeding. | None mentioned. | None mentioned. |

Data in the table above are based on the disseminations (published article, protocols or data from www.clinicaltrial.gov) from the trial in the relevant rows.
VKA – vitamin K antagonists, CT – computed tomography, INR – international normalized ratio, FFP – fresh frozen plasma, PCC – prothrombin complex concentrate, IU – international units.

## Supplementary references


1. Steiner T, Poli S, Griebe M, Husing J, Hajda J, Freiberger A, et al. Fresh frozen plasma versus prothrombin complex concentrate in patients with intracranial haemorrhage related to vitamin K antagonists (INCH): a randomised trial. Lancet Neurol. 2016;15:566-73.
2. Steiner T, Freiberger A, Griebe M, Husing J, Ivandic B, Kollmar R, et al. International normalised ratio normalisation in patients with coumarin-related intracranial haemorrhages--the INCH trial: a randomised controlled multicentre trial to compare safety and preliminary efficacy of fresh frozen plasma and prothrombin complex--study design and protocol. Int J Stroke. 2011;6:271-7.
3. Sarode R, Milling TJ, Jr., Refaai MA, Mangione A, Schneider A, Durn BL, Goldstein JN. Efficacy and safety of a 4-factor prothrombin complex concentrate in patients on vitamin K antagonists presenting with major bleeding: a randomized, plasma-controlled, phase IIIb study. Circulation. 2013;128:1234-43.
4. CSL Behring Clinical Research and Development. An open-label, randomized, multicenter Phase IIIb study to assess the efficacy, safety and tolerance of Beriplex® P/N compared with plasma for rapid reversal of coagulopathy induced by coumarin derivatives in subjects with acute major bleeding (BE1116_3002). Version 2.0, February 2012.
5. Boulis NM, Bobek MP, Schmaier A, Hoff JT. Use of factor IX complex in warfarin-related intracranial hemorrhage. Neurosurgery. 1999;45:1113-8; discussion 8-9.
6. Connolly SJ, Sharma M, Cohen AT, Demchuk AM, Czlonkowska A, Lindgren AG, et al. Andexanet for factor Xa inhibitor-associated acute intracerebral hemorrhage. N Engl J Med. 2024;390:1745-55.
7. Shadvar K, Sadaghi P, Hamishekar H, Mahmoodpoor A. Efficacy of prothrombin complex concentrate for reversal of major bleeding due to rivaroxaban: A pilot randomized controlled trial. J Clin Anesth. 2021;68:110093.
