# Supplementary material for: Prothrombin complex concentrate for reversal of oral anticoagulants in patients with oral anticoagulation-related critical bleeding: a systematic review of randomised clinical trials
Source: Scand J Trauma Resusc Emerg Med. 2025 Feb 4;33:19. doi: 10.1186/s13049-025-01334-1 (PMC11792222; doi:10.1186/s13049-025-01334-1)
Supplement: Supplementary file 5 — Additional file 5. [file 13049_2025_1334_MOESM5_ESM.pdf]

Additional file 5:

Supplement 6: Risk of bias assessment of included randomised clinical trials

Trial	Bias domain	Risk of bias	Argument
Steiner et al (2016) [1]	Allocation sequence generation	Low risk of bias	Reference to computer random number generator.<br><br>**Quote:** 'The allocation sequence was implemented with a randomisation list computer generated at the Coordination Centre for Clinical Trials at Heidelberg University Hospital (Heidelberg, Germany). The randomisation list linked sequential numbers to treatment codes allocated at random in site-stratified blocks of varying length (four, six, eight, and ten, with a probability 0.25 each) using a customised R program. ' [1]
	Allocation concealment	Low risk of bias	Sequentially numbered, opaque, sealed envelopes. Person generating randomization list was independent of investigators and further study procedures.<br><br>**Quote:** 'The person responsible for generation of the randomisation list was independent of all other trial procedures. Because of the emergency situation and the need to randomise without delay, every site was provided with a sufficient number of closed, non-transparent envelopes containing treatment allocation. Each envelope had an individual randomisation number on the outside. The investigators were instructed to assign the patient according to the envelope with the lowest randomisation number.' [1]
	Blinding of participants and treatment providers	High risk of bias	The trial utillized a prospective, randomized, open-label, blinded endpoint (PROBE) design. Blinding of the treating physician was not performed.<br><br>**Quote:** 'We did an investigator-initiated, multicentre, prospective, randomised, open-label, blinded-endpoint trial (PROBE design).' [1] 'Masking of treatment was not possible because of the different appearance of the two products and study drug preparation at bedside.' [1]
	Blinding of outcome assessors	Low risk of bias	As per the PROBE design the trial utillized observer-blinding for at least the laboratory data, radiological data and clinical outcomes. It is uncertain if personnel reporting and evaluating serious adverse events were blinded to the participants allocation.<br><br>**Quote:** 'Blinding of treatment will not be possible, because of the different appearance of the two products and preparations involved in the applications at bedside. However, the study is observer-blinded for all laboratory data (including the primary endpoint), and neuroradiological and clinical outcome assessment.' [2]
	Incomplete outcome data	Low risk of bias	Four participants were withdrawn after randomisation. Reasons for all attritions on the primary outcome measure were clearly documented. Multiple imputation was used as sensitivity analysis on primary outcome.<br><br>**Quote:** 'Four patients were withdrawn by the site investigator after randomization and before the beginning of infusion' [1] 'In any of these cases no further data were collected by the site investigator, and although these four patients technically were part of the full analysis data set, they remained excluded.' [1] 'A sensitivity analyses to check for the influence of these exclusions on the primary endpoint was carried out using multiple imputation with 50 replicates, where baseline and three hours INR measurement pairs were imputed based on the observes INR values. The resulting effect on the primary endpoint was estimated to be OR=29.5 [4.2; 206.7], p = 0.0007].' [1]

Trial	Bias domain	Risk of bias	Argument
Steiner et al (2016) [1]	Selective outcome reporting	Low risk of bias	A published protocol is available. All outcomes are reported as described in protocol. All expected outcomes are reported.
	Vested interest bias	High risk of bias	The trial is funded by an unrestricted grant from Octapharm AG, Lachen, Switzerland. Octapharm is the company behind Octaplex which is being used as the investigational medicinal product in the trial. The authors report that the company had no influence on the conduct or publication of the trial.<br><br>**Quote:** 'The funder of the study had no role in study design, data collection, data analysis, data interpretation, or writing of the report. The corresponding author had full access to all the data in the study and had final responsibility for the decision to submit for publication.' [1]
Sarode et al. (2013) [3]	Allocation sequence generation	Low risk of bias	Minimization was used to ensure balanced allocation stratified for site and bleeding type.<br><br>**Quote:** 'Patients were assigned by a centrally managed biased-coin minimization method, which controlled for balance in number of patients among treatment arms overall and per site as well as among bleeding type (comprising gastrointestinal, visible, intracranial hemorrhage, musculoskeletal, and other nonvisible bleeding).' [3]
	Allocation concealment	Low risk of bias	Centrally managed system making a breach of the allocation concealment unlikely.<br><br>**Quote:** Please see quote above.
	Blinding of participants and treatment providers	High risk of bias	The study used an open label design. Staff was not blinded for treatment allocation.<br><br>**Quote:** 'Study staff were not blinded to treatment allocation because of the inherent characteristics of the study drugs.' [3]
	Blinding of outcome assessors	Low risk of bias	Outcome assessors were blinded and method of blinding described.<br><br>**Quote:** 'Therefore, hemostatic efficacy was assessed by a blinded, independent Endpoint Adjudication Board (EAB). An independent Data Safety Monitoring Board reviewed unblinded data to assess patient safety. Serious adverse events (AEs) of interest to the Data Safety Monitoring Board (thromboembolic events, deaths, late bleeding events) were reviewed by a blinded, independent Safety Adjudication Board (SAB).' [3]
	Incomplete outcome data	Low risk of bias	Clean intention to treat analysis not performed as at least one patient was not analyzed in the group he was allocated to (allocated to PCC but did instead receive fresh frozen plasma). The patient was analyzed in the plasma group instead. Attritions seems balanced and not likely to be able to cause a shift in the conclusion of the trial.<br><br>**Quote:** 'Note that one patient assigned to 4F-PCC received plasma and was assigned to the plasma safety population' [3]
	Selective outcome reporting	Uncertain risk of bias	Protocol not available but www.clinicaltrials.gov registration is available (NCT00708435). Based on the information presented here it is uncertain if the wording of at least some of the outcome-measures was changed after completion of the trial recruitment phase (changes to outcomes submitted September 12, 2013).

**Trial**	**Bias domain**	**Risk of bias**	**Argument**
Sarode et al. (2013) [3]	Vested interest bias	High risk of bias	Sponsored by CSL Behring. CSL Behring is the company behind Beriplex P/N which was used as intervention treatment resulting in potential economic interest in the conclusion of the trial. Company employees were represented in the trial steering committee. Sponsor was responsible for data analysis and reporting of results.<br><br>**Quote:** 'This research was sponsored by CSL Behring. A steering committee of investigators, academic medical experts, and representatives of the sponsor oversaw the trial design and conduct with the assistance of the independent Data Safety Monitoring Board, SAB, and EAB. The sponsor participated in the selection of the board members. The sponsor was responsible for data processing, management, analysis of the data according to a predefined statistical analysis plan, and reporting of the results.' [3]
Boulis et al. (1999) [4]	Allocation sequence generation	Uncertain risk of bias	Allocation sequence generation not described. Trial only described randomizing patients to either fresh frozen plasma alone or fresh frozen plasma and factor IX complex concentrate.
	Allocation concealment	Uncertain risk of bias	Allocation concealment not described.
	Blinding of participants and treatment providers	High risk of bias	It is described that the treatment providers knew the allocation of the participant.<br><br>**Quote:** 'Although the present study was randomized, it was not blinded. The authors involved in serum sampling and organization of the delivery of plasma were aware of the study group of the patients' [4] 'Therefore, the bias that accompanies all unblinded studies must be considered when the present data are analyzed' [4]
	Blinding of outcome assessors	High risk of bias	It is described that the treatment providers knew the allocation of the participant, and that the trial was unblinded.<br><br>**Quote:** 'Although the present study was randomized, it was not blinded. The authors involved in serum sampling and organization of the delivery of plasma were aware of the study group of the patients' [4] 'Therefore, the bias that accompanies all unblinded studies must be considered when the present data are analyzed' [4]
	Incomplete outcome data	High risk of bias	The authors report an attrition of eight participants after randomisation. Four patients were withdrawn due to withdrawl-of-care orders and are highly likely to have died. Four participants (one allocated to factor IX complex concentrate and three to FFP) were excluded due to incomplete outcome data. Such a high and unbalanced attrition could potentially affect the conclusion of the trial.<br><br>**Quote:** 'These eight patients included two patients in the FIXCC- treated group and two patients in the FFP-treated group whose families requested withdrawal of support, because of neurological deterioration, before correction of coagulopathy. The remaining four patients (one from the FIXCC-treated group and three from the FFP-treated group) were excluded because of omissions in data collection (missed blood drawings) that made calculation of the time to correction impossible.' [4]
	Selective outcome reporting	Uncertain risk of bias	No protocol or similar prospective documentations were available. Trial not prospectively registered.
	Vested interest bias	Uncertain risk of bias	Financial source not described in the article.

**Trial**	**Bias domain**	**Risk of bias**	**Argument**
Shadvar et al. (2021) [5]	Allocation sequence generation	Uncertain risk of bias	Allocation sequence generation not described.
	Allocation concealment	Uncertain risk of bias	Allocation concealment not described.
	Blinding of participants and treatment providers	Uncertain risk of bias	Blinding of participants and treatment providers not described
	Blinding of outcome assessors	Uncertain risk of bias	Blinding of outcome assessors not described
	Incomplete outcome data	Uncertain risk of bias	It is unclear from the published report if there were any drop-outs or participants lost-to-follow-up
	Selective outcome reporting	Uncertain risk of bias	The online trial registration only mentions a single outcome.
	Vested interest bias	Uncertain risk of bias	Financial source not described in the article.
Connolly et al. (2024) [6]	Allocation sequence generation	Low risk of bias	The protocol clearly describes a randomised allocation scheme generated by sponsor<br><br>**Quote:** 'This study will be randomized 1:1 andexanet to usual care. Randomization will be stratified by site. The randomization scheme will be generated by the Sponsor.' [6]
	Allocation concealment	Low risk of bias	**Quote:** 'Patients will be randomized 1:1 to andexanet or usual care. Blocks will be implemented to ensure an equal number of patients from each treatment group within a block. The blocks will be assigned to each site. Only complete blocks will be assigned to the sites making the randomization functionally equivalent to a randomization schedule stratified by site. The block sizes will not be disclosed to the Investigators.' [6]
	Blinding of participants and treatment providers	High risk of bias	No blinding of local investigators or participants to the allocation was conducted<br><br>**Quote:** 'However, because of the many therapeutic options available to investigators to treat patients randomized to usual care, it is considered unfeasible to blind all study personnel (especially those directly involved in the care of an enrolled patient) to treatment assignment. Thus, the study allocation of each patient will be unblinded to the local investigator and most of the local site study team.' [6]
	Blinding of outcome assessors	Low risk of bias	Outcome assessors were blinded and method of blinding described.<br><br>**Quote:** 'To avoid bias, the study treatment assignment and anti-fXa activity levels will be blinded to the independent Endpoint Adjudication Committee and the Imaging Core Laboratory.' and 'One or more individuals at each site will be blinded to the treatment assignment and will be responsible to perform the neurologic assessments during the first 12 hours following randomization.' [6]

**Trial**	**Bias domain**	**Risk of bias**	**Argument**
Connolly et al. (2024) [6]	Incomplete outcome data	High risk of bias	The trial had a projected sample size of 900 participants. Efficacy was assessed at an interim-analysis involving 452 participants. This led to the premature stopping as andexanet alfa was assessed as superior to usual care. However, 530 participants were included in total, resulting in 78 participants not being included in the final efficacy analysis. This was, however, balanced between groups. In addition, 9 participants were unavailable for the complete primary outcome assessment for administrative reasons.<br><br>**Quote:** 'The interim analysis of the data from the first 452 patients enrolled was designated as the primary analysis of efficacy, whereas all 530 patients in the database were included in the safety analyses.' [6]
	Selective outcome reporting	Low risk of bias	All pre-specified overall outcomes accounted for. However, not all outcome analyses were broken down to andexanet alfa versus prothrombin complex concentrate.
	Vested interest bias	High risk of bias	The trial is sponsored by Alexion AstraZeneca Rare Disease, AstraZeneca Biopharmaceuticals. Alexion is the company behind andexanet alfa which is being used as the investigational medicinal product in the trial.<br><br>**Quote:** 'This trial was coordinated by the Population Health Research Institute, a joint institute of McMaster University and Hamilton Health Sciences, and was initially funded by Portola, which was purchased by Alexion AstraZeneca Rare Disease. Portola, followed by Alexion, was the trial sponsor and provided the andexanet free of charge.' [6]

Supplementary references

1. Steiner T, Poli S, Griebe M, Husing J, Hajda J, Freiberger A, et al. Fresh frozen plasma versus prothrombin complex concentrate in patients with intracranial haemorrhage related to vitamin K antagonists (INCH): a randomised trial. Lancet Neurol. 2016;15:566-73.
2. Steiner T, Freiberger A, Griebe M, Husing J, Ivandic B, Kollmar R, et al. International normalised ratio normalisation in patients with coumarin-related intracranial haemorrhages--the INCH trial: a randomised controlled multicentre trial to compare safety and preliminary efficacy of fresh frozen plasma and prothrombin complex--study design and protocol. Int J Stroke. 2011;6:271-7.
3. Sarode R, Milling TJ, Jr., Refaai MA, Mangione A, Schneider A, Durn BL, Goldstein JN. Efficacy and safety of a 4-factor prothrombin complex concentrate in patients on vitamin K antagonists presenting with major bleeding: a randomized, plasma-controlled, phase IIIb study. Circulation. 2013;128:1234-43.
4. Boulis NM, Bobek MP, Schmaier A, Hoff JT. Use of factor IX complex in warfarin-related intracranial hemorrhage. Neurosurgery. 1999;45:1113-8; discussion 8-9.
5. Shadvar K, Sadaghi P, Hamishekar H, Mahmoodpoor A. Efficacy of prothrombin complex concentrate for reversal of major bleeding due to rivaroxaban: A pilot randomized controlled trial. J Clin Anesth. 2021;68:110093.
6. Connolly SJ, Sharma M, Cohen AT, Demchuk AM, Czlonkowska A, Lindgren AG, et al. Andexanet for factor Xa inhibitor-associated acute intracerebral hemorrhage. N Engl J Med. 2024;390:1745-55.
