# Supplementary material for: Prothrombin complex concentrate for reversal of oral anticoagulants in patients with oral anticoagulation-related critical bleeding: a systematic review of randomised clinical trials
Source: Scand J Trauma Resusc Emerg Med. 2025 Feb 4;33:19. doi: 10.1186/s13049-025-01334-1 (PMC11792222; doi:10.1186/s13049-025-01334-1)
Supplement: Supplementary file 6 — Additional file 6 [file 13049_2025_1334_MOESM6_ESM.pdf]

Additional file 6:

Supplement 7: Definition and follow-up of each outcome in trials comparing PCC with fresh frozen plasma in participants with VKA-related bleeding

**Outcome**	**Trials / Data source for the outcome**	**Outcome reported in published articles?**	**Definition/scale used.**	**Primary, secondary or explorative outcome in trial**	**Follow-up period or day-of-assessment (after inclusion in trial).**	**Risk of bias – blinded assessment**	**Risk of bias – selective outcome reporting**	**Risk of bias – incomplete outcome data**
All-cause mortality	Steiner et al. (2016) [1]	Yes	NA.	Secondary	90 days	Low risk as endpoint assessor blinded for treatment allocation.	Low risk of bias, as all trials reported on this outcome.	Low risk of bias, as 'best-worst-case' and 'worst-best-case' did not change the conclusion.
	Sarode et al. (2013) [2, 3]	Yes	NA.	Secondary	45 days	Unclear risk as deaths were reported to an unblinded data safety monitoring committee but reviewed by an independent blinded safety adjudication board.		
Health-related quality of life	Steiner et al. (2016) [1]	Yes	European Quality of Life Questionnaire, five dimensions, five levels scale (EQ-5D-5L).	Secondary	Day-90	High risk as the trial utilised an open label design, and the health-related quality of life outcome was based on self-assessment.	Low risk of bias, as trial protocols or prospective trial registration was available for both trials.<br>.	NA.
	Sarode et al. (2013) [2, 3]	No.	NA.	NA.	NA.	NA.		

**Outcome**	**Trials / Data source for the outcome**	**Outcome reported in published articles?**	**Definition/scale used.**	**Primary, secondary or explorative outcome in trial**	**Follow-up period or day-of-assessment (after inclusion in trial).**	**Risk of bias – blinded assessment**	**Risk of bias – selective outcome reporting**	**Risk of bias – incomplete outcome data**
Serious adverse events	Steiner et al. (2016)	Yes	A serious AE (SAE) is any untoward medical occurrence that at any dose: Result in death, is life-threatening, requires or prolongs hospitalisation, results in persistent or significant disability, is a congenital abnormality, or another important medical event.	Secondary	90 days.	Unclear risk, as the trial utilised an open-label design, and it is unclear, if the treating physician was unblinded when detecting and reporting SAE.	Low risk of bias, as all trial reported on this outcome.	High risk, as ’best-worst-case’ and ’worst-best-case’ could change the conclusion.
	Sarode et al. (2013) [2, 3]	Yes	Any untoward medical occurrence that at any dose: resulted in death; was life-threatening; required in-patient hospitalization or prolongation of existing hospitalization; resulted in persistent or significant disability/incapacity; was a congenital anomaly/birth defect; was according to investigator’s judgement another medically important condition.	Secondary	45 days.	Unclear risk, as a blinded safety adjudication board is clearly described, when it comes to SAEs of interest to the Data Safety Monitoring Board. It is, however, uncertain, if the study-personal assessing and reporting SAEs to the safety adjudication board / trial database in general was blinded to the allocation of the participant.		

**Outcome**	**Trials / Data source for the outcome**	**Outcome reported in published articles?**	**Definition/scale used.**	**Primary, secondary or explorative outcome in trial**	**Follow-up period or day-of-assessment (after inclusion in trial).**	**Risk of bias – blinded assessment**	**Risk of bias – selective outcome reporting**	**Risk of bias – incomplete outcome data**
Poor functional outcome	Steiner et al. (2016) [1]	Yes	Modified Rankin Scale (mRS). Poor functional outcome defined as mRS 4-6.	Secondary	Day-90	Low risk, as blinding of outcome assessors is clearly stated and described.	Low risk of bias, as trial protocols or prospective trial registration was available for both trials.	Low risk of bias as 'best-worst-case' and 'worst-best-case' did not change the conclusion.
	Sarode et al. (2013) [2, 3]	Yes	Modified Rankin Scale (mRS). Good outcome defined as mRS <4. Poor functional outcome inferred from data as mRS 4-6.	Secondary	Day-45	Unclear risk, as the trial utilised an open-label design, and it is unclear, if the outcome adjudicator was blinded.		
Thromboembolic events	Steiner et al. (2016) [1]	Yes	Not directly described. However, thromboembolic events of special interest: Ischemic stroke (new signs on CT scans measured until day 15), myocardial infarction (typical laboratory and ECG signs), pulmonary embolism (confirmed by imaging methods and typical laboratory changes), deep vein thrombosis (confirmed by per-protocol ultrasound on day 3 or clinical ultrasound. In addition typical laboratory changes).	Secondary	90 days	Unclear risk, as the trial utilised an open-label design, and it is unclear, if the treating physician was blinded when assessing potential thromboembolic events in trial participants.	Low risk of bias, as all trial reported on this outcome.	High risk, as 'best-worst-case' and 'worst-best-case' could change the conclusion.

Outcome	Trials / Data source for the outcome	Outcome reported in published articles?	Definition/scale used.	Primary, secondary or explorative outcome in trial	Follow-up period or day-of-assessment (after inclusion in trial).	Risk of bias – blinded assessment	Risk of bias – selective outcome reporting	Risk of bias – incomplete outcome data
Thromboembolic events	Sarode et al. (2013) [2, 3]	Yes	No definition reported.	Unspecified	45 days	Unclear risk, as the trial utilised an open-label design, and it is unclear, if the study personnel diagnosing and reporting thromboembolic events were blinded. It is mentioned that the Safety adjudication Board reviewed reports of thromboembolic events in a blinded fashion, but their role is unclear.	Low risk of bias, as all trial reported on this outcome.	High risk, as 'best-worst-case' and 'worst-best-case' could change the conclusion.
Allergic reactions	Steiner et al. (2016) [1]	Yes	No definition reported.	Unspecified	90 days	Unclear risk, as the trial utilised an open-label design, and it is unclear, if the treating physician was blinded when assessing potential allergic reactions in trial participants.	Low risk of bias, as all trial reported on this outcome.	Low risk of bias, as 'best-worst-case' and 'worst-best-case' did not change the conclusion.

Outcome	Trials / Data source for the outcome	Outcome reported in published articles?	Definition/scale used.	Primary, secondary or explorative outcome in trial	Follow-up period or day-of-assessment (after inclusion in trial).	Risk of bias – blinded assessment	Risk of bias – selective outcome reporting	Risk of bias – incomplete outcome data
Allergic reactions	Sarode et al. (2013) [2, 3]	Yes	No definition reported.	Unspecified	45 days	Unclear risk, as the trial utilised an open-label design, and it is unclear, if the treating physician was blinded when assessing potential allergic reactions in trial participants.	Low risk of bias, as all trial reported on this outcome.	Low risk of bias, as 'best-worst-case' and 'worst-best-case' did not change the conclusion.
Pulmonary edema	Steiner et al. (2016) [1]	Yes	No definition reported.	Unspecified	90 days	Unclear risk, as the trial utilised an open-label design, and it is unclear, if the treating physician was blinded when assessing potential fluid overload in trial participants.	Low risk of bias, as all trials reported on this outcome.	Low risk of bias, as 'best-worst-case' and 'worst-best-case' did not change the conclusion.
	Sarode et al. (2013) [2, 3]	Yes	No definition reported.	Unspecified	45 days	Unclear risk, as the trial utilised an open-label design, and it is unclear, if the treating physician was blinded when assessing potential fluid overload in trial participants.		

**Outcome**	**Trials / Data source for the outcome**	**Outcome reported in published articles?**	**Definition/scale used.**	**Primary, secondary or explorative outcome in trial**	**Follow-up period or day-of-assessment (after inclusion in trial).**	**Risk of bias – blinded assessment**	**Risk of bias – selective outcome reporting**	**Risk of bias – incomplete outcome data**
Tardy INR correction	Steiner et al. (2016) [1]	Yes	INR >1.2 at 3 hours after start of treatment.	Primary	Three hours after start of treatment.<br><br>INR was also obtained 30 minutes after start of treatment.	Low risk of bias, as blinding of outcome assessment was clearly described.	Low risk of bias, as all trials reported on this outcome.	Low risk of bias, as 'best-worst-case' and 'worst-best-case' did not change the conclusion.
	Sarode et al. (2013) [2, 3]	Yes	INR >1.3 at 0.5 hours after end of infusion.	Primary	Measured 0.5 hours after end of infusion. INR was also measured at 0.5, 1, 3, 6, 12, and 24 hours after infusion started. We applied the sponsor for the 3-hour INR values. No data provided by the sponsor.	Low risk, as this outcome measure includes no subjective judgement. In addition, endpoint assessment was performed blinded to treatment allocation.		
Poor haemostatic efficacy	Steiner et al. (2016) [1]	Yes	Intracerebral haematoma volume increase of ≥33% on 24-hour cerebral CT compared to baseline CT.	Secondary	CT performed at hour-24 after start of study medication will be used to assess this outcome. In the trial, CT was performed at hour-3, hour-24 and at hour-72.	Low risk of bias, as blinded assessment of radiological outcomes is clearly described.	Low risk of bias, as all trials reported on this outcome.	High risk as, 'best-worst-case' and 'worst-best-case' could change the conclusion.

Outcome	Trials / Data source for the outcome	Outcome reported in published articles?	Definition/scale used.	Primary, secondary or explorative outcome in trial	Follow-up period or day-of-assessment (after inclusion in trial).	Risk of bias – blinded assessment	Risk of bias – selective outcome reporting	Risk of bias – incomplete outcome data
Poor haemostatic efficacy	Sarode et al. (2013) [2, 3]	Yes	Visible bleeding: Cessation of bleeding >4 hours after end of the infusion, and/or additional coagulation intervention required (e.g. plasma, whole blood cell pack, or coagulation factor products).<br>Musculoskeletal bleeding: no improvement by 4 hours after the end of infusion and/or the condition has deteriorated during the 24-hour period.<br>Intracranial haemorrhage: >35% increase in haematoma volume compared to baseline on repeat CT scan performed at the 24 hour time point.<br>Non-visible bleeding that is not listed above: >20% decrease in both Hb/HCT at 24 hours compared to baseline (initial correction of decrease in haemoglobin with PRBCs, with a transfusion trigger of a Hb ≤8 ±1 g/dL.	Primary	Please see definition.	Low risk of bias, as blinding of Endpoint Adjudication Board is clearly stated.	Low risk of bias, as all trials reported on this outcome.	High risk, as 'best-worst-case' and 'worst-best-case' could change the conclusion.

**Outcome**	**Trials / Data source for the outcome**	**Outcome reported in published articles?**	**Definition/scale used.**	**Primary, secondary or explorative outcome in trial**	**Follow-up period or day-of-assessment (after inclusion in trial).**	**Risk of bias – blinded assessment**	**Risk of bias – selective outcome reporting**	**Risk of bias – incomplete outcome data**
Transfusion of red blood cells	Steiner et al. (2016) [1]	No	NA.	NA.	NA.	NA.	Low risk of bias. Only one trial reports on this outcome, however, the other trials include only intracranial haemorrhage, and transfusions of red blood cells are rarely used for treatment of intracranial haemorrhage.	Low risk of bias, as 'best-worst-case' and 'worst-best-case' did not change the conclusion.
	Sarode et al. (2013) [2, 3]	Yes	Patients receiving ≥1 transfusion with packed red blood cells.	Secondary	Not specified.	Unclear as the trial utilised an open-label design, and it is unclear, if the treating physician was blinded.		

All data in the table above are based on the disseminations (published article, protocols or data from www.clinicaltrial.gov) from the trial in the relevant row
NA. – not applicable, HRQoL – health-related quality of life, AE – adverse events, SAE -serious adverse events, ICH-GCP – international conference on harmonisation good clinical practice, FIXCC – factor IX complex concentrate, mRS – modified Rankin scale, CT – computed tomography, ECG – electrocardiogram, Hb – haemoglobin, HCT – haematocrit, PRBC – packed red blood cells.

Supplement 8: Definition and follow-up of each outcome in trials comparing PCC plus fresh frozen plasma with fresh frozen plasma alone in participants with VKA-related bleeding

**Outcome**	**Trials**	**Outcome reported in published articles?**	**Definition/scale used.**	**Primary, secondary or explorative outcome in trial**	**Follow-up period or day-of-assessment (after inclusion in trial).**	**Risk of bias – blinded assessment**	**Risk of bias – selective outcome reporting**	**Risk of bias – incomplete outcome data**
All-cause mortality	Boulis et al. (1999) [4]	Yes	NA.	Unspecified	Recorded during admission.	High risk as outcome assessor was not blinded to treatment allocation.	Low risk of bias, as all trials reported on this outcome.	Low risk of bias, as 'best-worst-case' and 'worst-best-case' did not change the conclusion.
Health-related quality-of-life	Boulis et al. (1999) [4]	No.	NA.	NA.	NA.	NA.	Unclear risk of bias, as trial-protocol was unavailable.	NA.

**Outcome**	**Trials**	**Outcome reported in published articles?**	**Definition/scale used.**	**Primary, secondary or explorative outcome in trial**	**Follow-up period or day-of-assessment (after inclusion in trial).**	**Risk of bias – blinded assessment**	**Risk of bias – selective outcome reporting**	**Risk of bias – incomplete outcome data**
Serious adverse events	Boulis et al. (1999) [4]	Yes	Not reported. For the purpose of this review we extracted the number of serious adverse events by applying the ICH-GCP definition on the list of "complications" listed in the article.<br><br>FIXCC group: 2 deaths (before intervention), no additional complications documented (table 1)<br><br>No treatment group: 2 deaths (before intervention). 3 death after intervention. Two additional participants - one with MI and one with pulmonary edema (table 1). Supraventricular tachyarrythmia was not considered an SAE.	Unspecified	Recorded during admission.	High risk of bias, as the trial was unblinded.	Low risk of bias, as all trial reported on this outcome.	Low risk of bias, as 'best-worst-case' and 'worst-best-case' did not change the conclusion.
Poor functional outcome	Boulis et al. (1999) [4]	No	NA.	NA.	NA.	NA.	Unclear risk of bias, as trial-protocol was unavailable.	NA.

**Outcome**	**Trials**	**Outcome reported in published articles?**	**Definition/scale used.**	**Primary, secondary or explorative outcome in trial**	**Follow-up period or day-of-assessment (after inclusion in trial).**	**Risk of bias – blinded assessment**	**Risk of bias – selective outcome reporting**	**Risk of bias – incomplete outcome data**
Thromboembolic events	Boulis et al. (1999) [4]	Yes	No definition reported.	Unspecified	Recorded during admission.	High risk of bias, as the trial was unblinded.	Low risk of bias, as all trial reported on this outcome.	Low risk of bias, as 'best-worst-case' and 'worst-best-case' did not change the conclusion.
Allergic reactions	Boulis et al. (1999) [4]	No	NA.	NA.	NA.	NA.	Unclear risk of bias, as trial-protocol was unavailable.<br><br>It is unclear, if the reason is for this outcome not being reported was that no allergic reactions occurred during the conduct of the trial.	NA.
Pulmonary edema	Boulis et al. (1999) [4]	Yes	No definition reported.	Unspecified	Recorded during admission.	High risk of bias, as the trial was unblinded.	Low risk of bias, as all trials reported on this outcome.	Low risk of bias, as 'best-worst-case' and 'worst-best-case' did not change the conclusion.

Outcome	Trials	Outcome reported in published articles?	Definition/scale used.	Primary, secondary or explorative outcome in trial	Follow-up period or day-of-assessment (after inclusion in trial).	Risk of bias – blinded assessment	Risk of bias – selective outcome reporting	Risk of bias – incomplete outcome data
Tardy INR correction	Boulis et al. (1999) [4]	Yes<br><br>Data not presented in a form allowing it to be included in the meta-analysis.	Trial used INR of 1.3 as goal of correction.	Unspecified	INR tested at 2, 4, 6, 8, 10 and 14 hours after enrolment.	Low risk, as this outcome measure includes no subjective judgement.	Low risk of bias, as all trials reported on this outcome.	NA.
Poor haemostatic efficacy	Boulis et al. (1999) [4]	No	NA.	NA.	NA.	NA.	Unclear risk of bias, as trial-protocol was unavailable.	NA.
Transfusion of red blood cells	Boulis et al. (1999) [4]	No	NA.	NA.	NA.	NA.	Low risk of bias. This trial includes only participants with intracranial haemorrhage, and transfusions of red blood cells is rarely used for treatment of intracranial haemorrhage.	NA.

All data in the table above are based on the disseminations (published article, protocols or data from www.clinicaltrial.gov) from the trial in the relevant row
NA. – not applicable, SAE -serious adverse events, ICH-GCP – international conference on harmonisation good clinical practice, FIXCC – factor IX complex concentrate, INR – international normalised ratio, MI – myocardial infarction.

Supplement 9: Definition and follow-up of each outcome in trials comparing PCC with fresh frozen plasma alone in participants with Factor Xa-related bleeding

**Outcome**	**Trials**	**Outcome reported in published articles?**	**Definition/scale used.**	**Primary, secondary or explorative outcome in trial**	**Follow-up period or day-of-assessment (after inclusion in trial).**	**Risk of bias – blinded assessment**	**Risk of bias – selective outcome reporting**	**Risk of bias – incomplete outcome data**
All-cause mortality	Shadvar et al. (2021) [5]	Yes	NA.	Unspecified	Mortality at 30 days	Unclear risk of bias, as blinding was not described	Low risk of bias, as all trials reported on this outcome.	Low risk of bias, as 'best-worst-case' and 'worst-best-case' did not change the conclusion.
Health-related quality-of-life	Shadvar et al. (2021) [5]	No.	NA.	NA.	NA.	NA.	Unclear risk of bias, as trial-protocol was unavailable.	NA.
Serious adverse events	Shadvar et al. (2021) [5]	No	Not reported. The article does mention the number of participants suffering acute kidney injury and thrombotic complications.	NA.	NA.	NA.	Unclear risk of bias, as trial-protocol was unavailable.	NA.
Poor functional outcome	Shadvar et al. (2021) [5]	No	NA.	NA.	NA.	NA.	Unclear risk of bias, as trial-protocol was unavailable.	NA.
Thromboembolic events	Shadvar et al. (2021) [5]	Yes	No definition reported.	Unspecified	Unspecified	Unclear risk of bias, as blinding was not described	Low risk of bias, as all trials reported on this outcome.	Low risk of bias, as 'best-worst-case' and 'worst-best-case' did not change the conclusion.

Outcome	Trials	Outcome reported in published articles?	Definition/scale used.	Primary, secondary or explorative outcome in trial	Follow-up period or day-of-assessment (after inclusion in trial).	Risk of bias – blinded assessment	Risk of bias – selective outcome reporting	Risk of bias – incomplete outcome data
Allergic reactions	Shadvar et al. (2021) [5]	No	NA.	NA.	NA.	NA.	Unclear risk of bias, as trial-protocol was unavailable.	NA.
Pulmonary edema	Shadvar et al. (2021) [5]	No	NA.	NA.	NA.	NA.	Unclear risk of bias, as trial-protocol was unavailable.	NA.
Tardy INR correction	Shadvar et al. (2021) [5]	No	NA.	NA.	NA.	NA.	Low risk as INR measurements are not used for patients with bleeding related to DOAC	NA.
Poor haemostatic efficacy	Shadvar et al. (2021) [5]	No	NA.	NA.	NA.	NA.	Unclear risk of bias, as trial-protocol was unavailable.	NA.
Transfusion of red blood cells	Shadvar et al. (2021) [5]	No	NA.	NA.	NA.	NA.	Unclear risk of bias, as trial-protocol was unavailable.	NA.

All data in the table above are based on the disseminations (published article, protocols or data from www.clinicaltrial.gov) from the trial in the relevant row
NA. – not applicable, INR – international normalised ratio, DOAC – direct oral anticoagulant.

Supplement 10: Definition and follow-up of each outcome in trials comparing PCC with andexanet alfa in participants with Factor Xa-related bleeding.

**Outcome**	**Trials**	**Outcome reported in published articles?**	**Definition/scale used.**	**Primary, secondary or explorative outcome in trial**	**Follow-up period or day-of-assessment (after inclusion in trial).**	**Risk of bias – blinded assessment**	**Risk of bias – selective outcome reporting**	**Risk of bias – incomplete outcome data**
All-cause mortality	Connolly et al. [6]	No – reported for andexanet versus usual care but not for andexanet versus PCC	NA.	Secondary	30 days	NA.	High risk of bias as the data exists but not reported.	NA.
Health-related quality-of-life	Connolly et al. [6]	No.	European Quality of Life Questionnaire, five dimensions scale	Secondary	30 days	NA.	High risk of bias as the data exists but not reported.	NA.
Serious adverse events	Connolly et al. [6]	No	An SAE is any untoward medical occurrence that at any dose: Result in death, is life-threatening, requires or prolongs hospitalisation, results in persistent or significant disability, is a congenital abnormality, or another important medical event.	NA.	NA.	NA.	Unclear risk of bias as the data exists but it was not prospectively declared to report on this outcome.	NA.
Poor functional outcome	Connolly et al. [6]	No – reported for andexanet versus usual care but not for andexanet versus PCC	Modified Rankin Scale (mRS). Poor functional outcome defined as mRS 4-6.	Secondary	30 days	NA.	High risk of bias as the data exists but not reported.	NA.
Thromboembolic events	Connolly et al. [6]	No – reported for andexanet versus usual care but not for andexanet versus PCC	No definition reported.	Secondary	30 days	NA.	High risk of bias as the data exists but not reported.	NA.

Outcome	Trials	Outcome reported in published articles?	Definition/scale used.	Primary, secondary or explorative outcome in trial	Follow-up period or day-of-assessment (after inclusion in trial).	Risk of bias – blinded assessment	Risk of bias – selective outcome reporting	Risk of bias – incomplete outcome data
Allergic reactions	Connolly et al. [6]	No.	NA.	NA.	NA.	NA.	Unclear risk of bias as the data exists but it was not prospectively declared to report on this outcome.	NA.
Pulmonary edema	Connolly et al. [6]	No.	NA.	NA.	NA.	NA.	Unclear risk of bias as the data exists but it was not prospectively declared to report on this outcome.	NA.
Tardy INR correction	Connolly et al. [6]	No	NA.	NA.	NA.	NA.	Low risk as INR measurements are not used for patients with bleeding related to DOAC	NA.
Poor haemostatic efficacy	Connolly et al. [6]	Yes	Poor haemostatic efficacy was defined as any of the following: a change in the hematoma volume >35% within 12 hours after baseline, an increase in the NIHSS score of ≥7 points at 12 hours, or receipt of rescue therapies such as andexanet, prothrombin complex concentrate, or surgery to decompress the hematoma within 3 to 12 hours after randomization.	Primary	12 hours	Low risk, endpoint assessment was performed blinded to treatment allocation	Low risk	High risk, as 'best-worst-case' and 'worst-best-case' could change the conclusion

**Outcome**	**Trials**	**Outcome reported in published articles?**	**Definition/scale used.**	**Primary, secondary or explorative outcome in trial**	**Follow-up period or day-of-assessment (after inclusion in trial).**	**Risk of bias – blinded assessment**	**Risk of bias – selective outcome reporting**	**Risk of bias – incomplete outcome data**
Transfusion of red blood cells	Connolly et al. [6]	No	NA.	NA.	NA.	NA.	Low risk of bias. This trial includes only participants with intracranial haemorrhage, and transfusions of red blood cells is rarely used for treatment of intracranial haemorrhage.	NA.

All data in the table above are based on the disseminations (published article, protocols or data from www.clinicaltrial.gov) from the trial in the relevant row
NA. – not applicable, SAE -serious adverse events, mRS – modified Rankin scale, DOAC – direct oral anticoagulant, NIHSS – National Institute of Health Stroke Scale.

Supplementary references

1. Steiner T, Poli S, Griebe M, Husing J, Hajda J, Freiberger A, et al. Fresh frozen plasma versus prothrombin complex concentrate in patients with intracranial haemorrhage related to vitamin K antagonists (INCH): a randomised trial. Lancet Neurol. 2016;15:566-73.
2. CSL Behring Clinical Research and Development. An open-label, randomized, multicenter Phase IIIb study to assess the efficacy, safety and tolerance of Beriplex® P/N compared with plasma for rapid reversal of coagulopathy induced by coumarin derivatives in subjects with acute major bleeding (BE1116_3002). Version 2.0, February 2012.
3. Sarode R, Milling TJ, Jr., Refaai MA, Mangione A, Schneider A, Durn BL, Goldstein JN. Efficacy and safety of a 4-factor prothrombin complex concentrate in patients on vitamin K antagonists presenting with major bleeding: a randomized, plasma-controlled, phase IIIb study. Circulation. 2013;128:1234-43.
4. Boulis NM, Bobek MP, Schmaier A, Hoff JT. Use of factor IX complex in warfarin-related intracranial hemorrhage. Neurosurgery. 1999;45:1113-8; discussion 8-9.
5. Shadvar K, Sadaghi P, Hamishekar H, Mahmoodpoor A. Efficacy of prothrombin complex concentrate for reversal of major bleeding due to rivaroxaban: A pilot randomized controlled trial. J Clin Anesth. 2021;68:110093.
6. Connolly SJ, Sharma M, Cohen AT, Demchuk AM, Czlonkowska A, Lindgren AG, et al. Andexanet for factor Xa inhibitor-associated acute intracerebral hemorrhage. N Engl J Med. 2024;390:1745-55.
