# Supplementary material for: Prothrombin complex concentrate for reversal of oral anticoagulants in patients with oral anticoagulation-related critical bleeding: a systematic review of randomised clinical trials
Source: Scand J Trauma Resusc Emerg Med. 2025 Feb 4;33:19. doi: 10.1186/s13049-025-01334-1 (PMC11792222; doi:10.1186/s13049-025-01334-1)
Supplement: Supplementary file 7 — Additional file 7. [file 13049_2025_1334_MOESM7_ESM.pdf]

Additional file 7:

Supplement 11: Sensitivity analysis - 'best-worst-case' 'worst-best-case' scenario – PCC versus fresh frozen plasma in participants with VKA-related bleeding

Supplementary table 1: Sensitivity analysis - 'best-worst-case' 'worst-best-case' scenario – PCC versus fresh frozen plasma in participants with VKA-related bleeding (categorical outcomes)

	No. of	No. of events and participants		Risk ratio (95% CI)*	
	trials	PCC	FFP	Fixed effect	Random effects
**All-cause mortality**					
'Best-worst'	2	15/135	16/135	0.91 (0.48 to 1.73)	0.91 (0.19 to 4.25)
'Worst-best'	2	20/135	13/135	1.50 (0.79 to 2.85)	1.39 (0.35 to 5.54)
**Serious adverse events**					
'Best-worst'	2	48/135	39/135	1.22 (0.87 to 1.71)	1.20 (0.86 to 1.68)
'Worst-best'	2	53/135	36/135	1.46 (1.03 to 2.06)	1.47 (1.04 to 2.07)
**Poor functional outcome**					
'Best-worst'	2	20/40	22/38	0.86 (0.58 to 1.27)	0.89 (0.60 to 1.30)
'Worst-best'	2	24/40	16/38	1.41 (0.91 to 2.19)	1.48 (0.69 to 3.20)
**Thromboembolic events**					
'Best-worst'	2	15/135	12/135	1.22 (0.60 to 2.48)	1.23 (0.61 to 2.48)
'Worst-best'	2	20/135	9/135	2.20 (1.04 to 4.66)	2.15 (1.00 to 4.60)
**Allergic reactions**					
'Best-worst'	2	0/135	5/135	0.18 (0.02 to 1.49)	Insufficient data
'Worst-best'	2	5/135	2/135	2.47 (0.49 to 12.35)	2.29 (0.42 to 12.51)
**Pulmonary edema**					
'Best-worst'	2	2/135	7/135	0.29 (0.06 to 1.33)	0.36 (0.09 to 1.56)
'Worst-best'	2	7/135	4/135	1.77 (0.53 to 5.92)	1.66 (0.52 to 5.24)
**Tardy INR correction**					
'Best-worst'	2	46/135	123/135	0.37 (0.29 to 0.48)	0.37 (0.29 to 0.48)
'Worst-best'	2	56/135	115/135	0.49 (0.39 to 0.60)	0.49 (0.40 to 0.60)
**Poor haemostatic efficacy**					
'Best-worst'	2	35/135	59/135	0.59 (0.42 to 0.83)	0.56 (0.36 to 0.89)
'Worst-best'	2	45/135	48/135	0.94 (0.67 to 1.30)	0.93 (0.67 to 1.30)
**Transfusion of red blood cells**					
'Best-worst'	1	48/107	52/109	0.94 (0.71 to 1.25)	Insufficient data
'Worst-best'	1	57/107	47/109	1.24 (0.93 to 1.63)	Insufficient data

No. – Number, PCC – prothrombin complex concentrate, FFP – fresh frozen plasma, CI – confidence intervals
In the 'best-worst' scenario participants with missing outcome data in the PCC group will be assumed not to have suffered the event, and participants in the FFP group will be assumed to have suffered the event. In the 'worst-best' scenario patient with missing outcome data in the PCC group will be assumed to have suffered the event, and participants in the FFP group will be assumed not to have suffered the event.
* Effect estimates below 1.00 favour treatment.

Supplementary table 2: Sensitivity analysis - 'best-worst-case' 'worst-best-case' scenario – PCC versus fresh frozen plasma in participants with VKA-related bleeding (continuous outcomes)

	No. of trials	Mean (standard deviation) [no. of participants]		Fixed effect risk ratio (95% CI)*
		PCC	FFP	
**Health-related quality of life**				
'Best-worst'	1	9.97 (2.75) [23]	6.88 (2.99) [18]	3.09 (1.31 to 4.87)
'Worst-best'	1	8.53 (2.75) [23]	9.54 (2.99) [18]	-1.01 (-2.79 to 0.77)

No. – Number, PCC – prothrombin complex concentrate, FFP – fresh frozen plasma, CI – confidence intervals
In the 'best-worst' scenario participants with missing outcome data in the PCC group will contribute the group mean plus two standard deviations of the group mean, and participants in the FFP group will contribute the group mean minus two standard deviations of the group mean. In the 'worst-best' scenario participants with missing outcome data in the PCC group will contribute the group mean minus two standard deviations of the group mean, and participants in the FFP group will contribute the group mean plus two standard deviations of the group mean.
*Effect estimate above 0.00 favour treatment.

Supplement 12: Sensitivity analysis - 'best-worst-case' 'worst-best-case' scenario – PCC plus fresh frozen plasma versus fresh frozen plasma alone in participants with VKA-related bleeding

Supplementary table 3: Sensitivity analysis - 'best-worst-case' 'worst-best-case' scenario – PCC plus fresh frozen plasma versus fresh frozen plasma alone in participants with VKA-related bleeding (categorical outcomes)

	No. of trials	No. of events and participants		Fixed effect risk ratio (95% CI)*
		PCC plus FFP	FFP alone	
**All-cause mortality**				
'Best-worst'	1	2/8	5/13	0.65 (0.16 to 2.59)
'Worst-best'	1	2/8	5/13	0.65 (0.16 to 2.59)
**Serious adverse events**				
'Best-worst'	1	2/8	10/13	0.33 (0.09 to 1.12)
'Worst-best'	1	3/8	7/13	0.70 (0.25 to 1.94)
**Thromboembolic events**				
'Best-worst'	1	0/8	6/13	0.12 (0.01 to 1.88)
'Worst-best'	1	3/8	1/13	4.88 (0.61 to 39.21)
**Pulmonary edema**				
'Best-worst'	1	0/8	6/13	0.12 (0.01 to 1.88)
'Worst-best'	1	3/8	1/13	4.88 (0.61 to 39.21)

No. – Number, PCC – prothrombin complex concentrate, FFP – fresh frozen plasma, CI – confidence intervals

In the 'best-worst' scenario participants with missing outcome data in the PCC plus FFP group will be assumed not to have suffered the event, and participants in the FFP alone group will be assumed to have suffered the event. In the 'worst-best' scenario patient with missing outcome data in the PCC plus FFP will be assumed to have suffered the event, and participants in the FFP alone group will be assumed not to have suffered the event.

* Effect estimates below 1.00 favour treatment.

Supplement 13: Sensitivity analysis - 'best-worst-case' 'worst-best-case' scenario – PCC versus fresh frozen plasma in participants with factor Xa-related bleeding

Supplementary table 4: Sensitivity analysis - 'best-worst-case' 'worst-best-case' scenario – PCC versus fresh frozen plasma in participants with factor Xa-related bleeding (chategorical outcomes)

	No. of trials	No. of events and participants		Fixed effect risk ratio (95% CI)*
		PCC	FFP	
**All-cause mortality**				
'Best-worst'	1	3/20	4/21	0.79 (0.20 to 3.09)
'Worst-best'	1	3/20	4/21	0.79 (0.20 to 3.09)
**Thromboembolic events**				
'Best-worst'	1	1/20	0/21	3.14 (0.14 to 72.92)
'Worst-best'	1	1/20	0/21	3.14 (0.14 to 72.92)

No. – Number, PCC – prothrombin complex concentrate, FFP – fresh frozen plasma, CI – confidence intervals

In the 'best-worst' scenario participants with missing outcome data in the PCC group will be assumed not to have suffered the event, and participants in the FFP group will be assumed to have suffered the event. In the 'worst-best' scenario patient with missing outcome data in the PCC group will be assumed to have suffered the event, and participants in the FFP group will be assumed not to have suffered the event.

* Effect estimates below 1.00 favour treatment.

Supplement 14: Sensitivity analysis - 'best-worst-case' 'worst-best-case' scenario – PCC versus andexanet alfa in participants with factor Xa-related bleeding

Supplementary table 5: Sensitivity analysis - 'best-worst-case' 'worst-best-case' scenario – PCC versus andexanet alfa in participants with factor Xa-related bleeding (chategorical outcomes)

	No. of trials	No. of events and participants		Fixed effect risk ratio (95% CI)*
		PCC	Andexanet alfa	
**Poor haemostatic efficacy**				
'Best-worst'	1	94/230	113/263	0.95 (0.77 to 1.17)
'Worst-best'	1	129/230	74/263	1.99 (1.59 to 2.49)

No. – Number, PCC – prothrombin complex concentrate, CI – confidence intervals
In the 'best-worst' scenario participants with missing outcome data in the PCC group will be assumed not to have suffered the event, and participants in the andexanet alfa group will be assumed to have suffered the event. In the 'worst-best' scenario patient with missing outcome data in the PCC group will be assumed to have suffered the event, and participants in the andexanet alfa group will be assumed not to have suffered the event.
* Effect estimates below 1.00 favour treatment.
