# Supplementary material for: Prothrombin complex concentrate for reversal of oral anticoagulants in patients with oral anticoagulation-related critical bleeding: a systematic review of randomised clinical trials
Source: Scand J Trauma Resusc Emerg Med. 2025 Feb 4;33:19. doi: 10.1186/s13049-025-01334-1 (PMC11792222; doi:10.1186/s13049-025-01334-1)
Supplement: Supplementary file 8 — Additional file 8. [file 13049_2025_1334_MOESM8_ESM.pdf]

Additional file 8

Supplement 15: Beta-binominal regression

Among trials using fresh frozen plasma as control treatment we attempted to fit beta-binominal regression to the outcomes; pulmonary oedema and allergic reactions. This was done as they contained zero-events in one or both treatment arms. However, the regression analysis for pulmonary oedema did not converge and the regression analysis for allergic reactions yielded non-sensical results. This was probably caused by small amount of trials.
The trial using PCC as add-on to fresh frozen plasma (Boulis et al.) contained zero event categories in the outcomes; thromboembolic events and in pulmonary oedema. The regression analysis for thromboembolic events did not converge and the regression analysis for pulmonary oedema yielded non-sensical results.
