# Supplementary material for: Prothrombin complex concentrate for reversal of oral anticoagulants in patients with oral anticoagulation-related critical bleeding: a systematic review of randomised clinical trials
Source: Scand J Trauma Resusc Emerg Med. 2025 Feb 4;33:19. doi: 10.1186/s13049-025-01334-1 (PMC11792222; doi:10.1186/s13049-025-01334-1)
Supplement: Supplementary file 9 — Additional file 9. [file 13049_2025_1334_MOESM9_ESM.pdf]

Additional file 9:

Supplement 16: Trial Sequential Analysis

Supplementary table 6: Results of Trial Sequential Analysis of PCC versus fresh frozen plasma

Outcome	No. of trials	RRR	Pc	D^2	DARIS*	% of DARIS obtained	TSA boundaries crossed? Superiority boundaries	Futility boundaries
**Primary outcomes**								
All-cause mortality	2	20%	9.8%	73%	43,928	0.6%	No	No
Serious adverse events	2	20%	27.3%	0%	3552	7.4%	No	No
**Secondary outcomes**								
Poor functional outcome	2	20%	50.0%	0%	1414	4.8%	No	No
Thromboembolism	2	20%	6.8%	1%	17973	1.5%	No	No
Allergic reactions	2	20%	1.5%	0%	84540	0.3%	No	No
**Explorative outcomes**								
Tardy INR correction	2	20%	90.6%	0%	262	96.2%	Yes	NA
Poor haemostatic efficacy	2	20%	38.7%	36%	3358	7.4%	No	No

No. – number; RRR – assumed relative risk reduction (dichotomous outcomes); Pc – Proportion in control group with outcome (dichotomous outcomes); D^2 – Diversity; DARIS – Diversity adjusted required information size; TSA – Trial sequential analysis.

*α-level (type 1 error risk) of 1.25% and a β-level (type 2 error risk) of 90% used in all calculation of DARIS.

Supplementary figure 1: Trial Sequential Analysis - Tardy INR correction

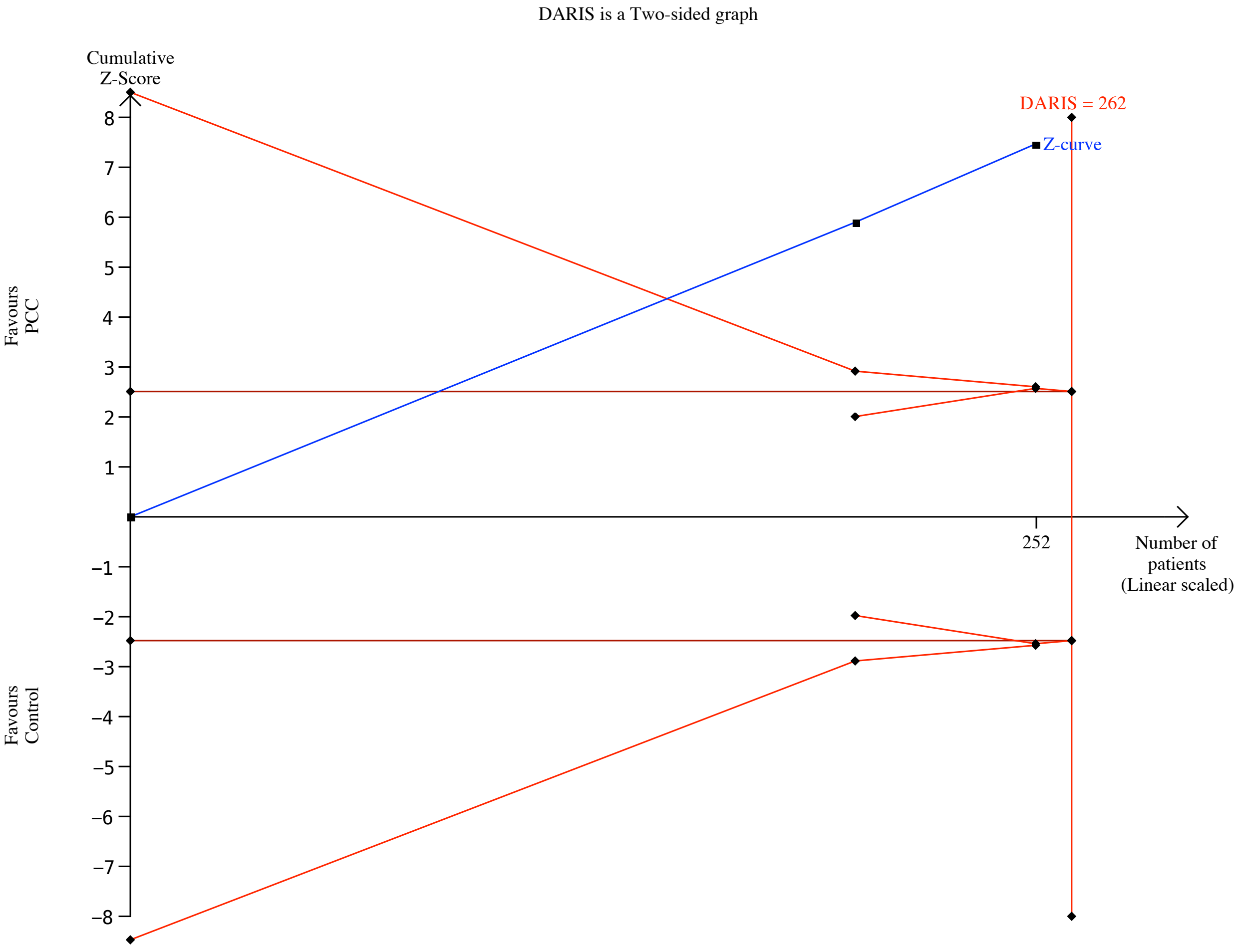

DARIS – diversity adjusted required information size, PCC – prothrombin complex concentrate, INR – international normalized ratio.

Cumulative Z-value obtained from random-effects meta-analysis. We see that the Lan-DeMets monitoring boundaries are crossed indicating superiority of the PCC in correcting INR. DARIS estimated to 262 based on a projected 20% relative risk reduction, an incidence in the control arm of 90.6%, a type 1 error of 1.25%, a type 2 error of 10% and a diversity (heterogeneity) (D^2) of 0%.
