# Supplementary material for: Prothrombin complex concentrate for reversal of oral anticoagulants in patients with oral anticoagulation-related critical bleeding: a systematic review of randomised clinical trials
Source: Scand J Trauma Resusc Emerg Med. 2025 Feb 4;33:19. doi: 10.1186/s13049-025-01334-1 (PMC11792222; doi:10.1186/s13049-025-01334-1)
Supplement: Supplementary file 10 — Additional file 10. [file 13049_2025_1334_MOESM10_ESM.pdf]

# Additional file 10:

## Supplement 17: Subgroup analysis - participants with intracranial haemorrhage compared to participants with other types of bleeding among participants with VKA-related bleeding

### Supplementary figure 2: subgroup analysis – mortality

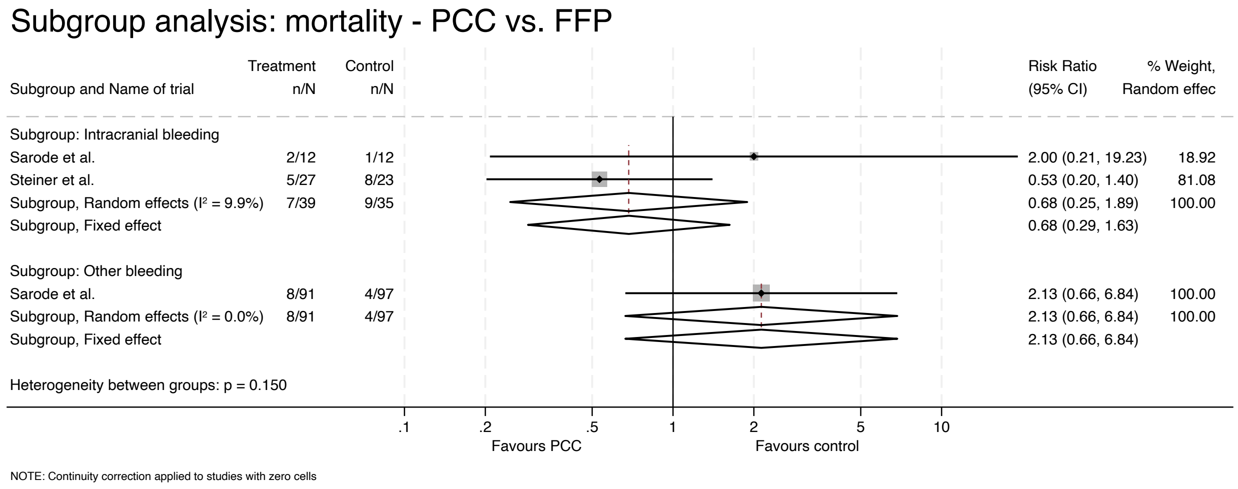


RR – relative risk, CI – confidence intervals, PCC – prothrombin complex concentrate, FFP – fresh frozen plasma

### Supplementary figure 3: subgroup analysis – serious adverse events

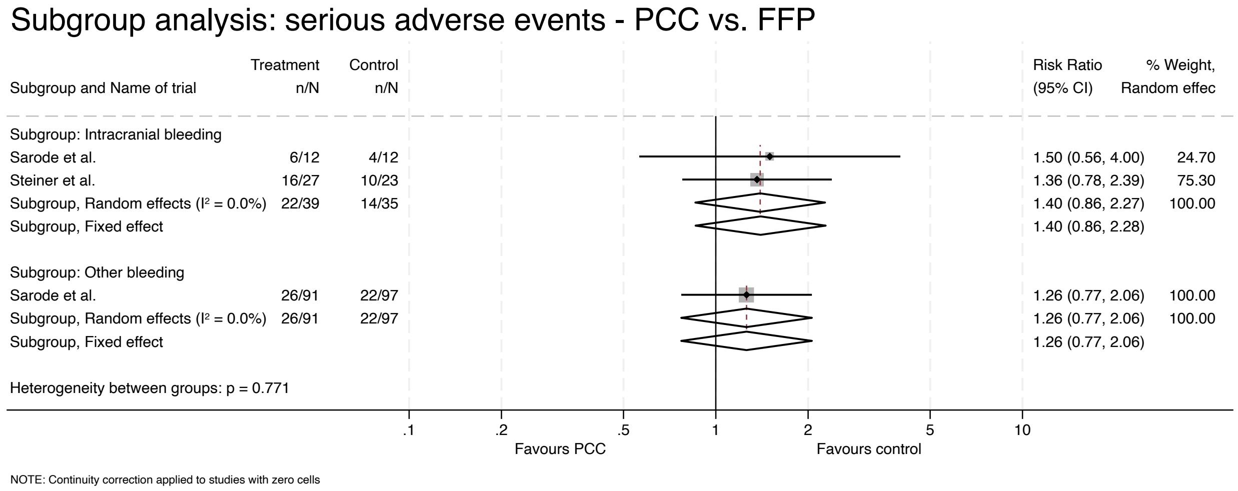


RR – relative risk, CI – confidence intervals, PCC – prothrombin complex concentrate, FFP – fresh frozen plasma

**Supplementary figure 4: subgroup analysis – thromboembolic events**

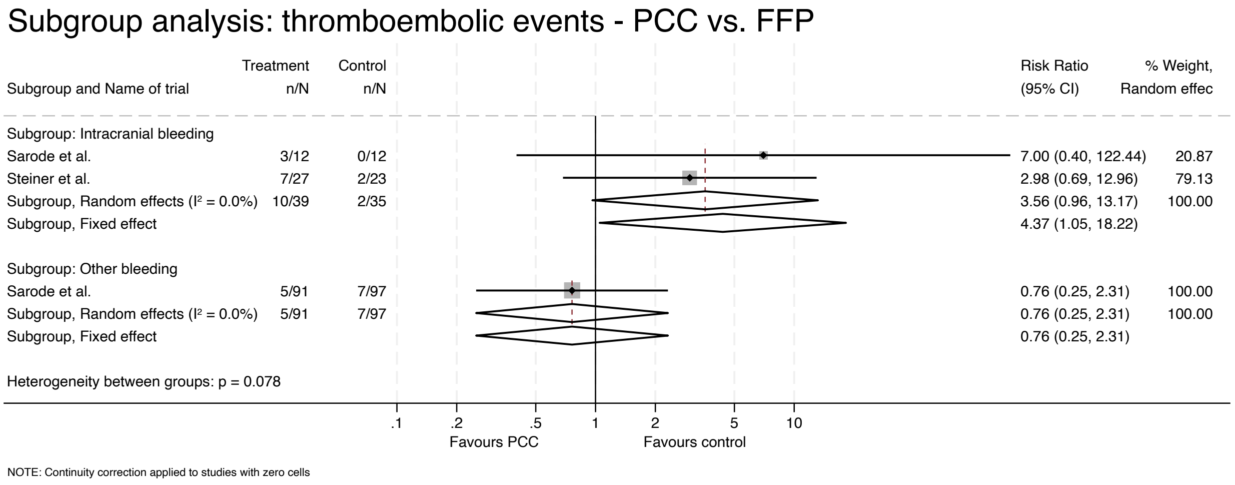


RR – relative risk, CI – confidence intervals, PCC – prothrombin complex concentrate, FFP – fresh frozen plasma

**Supplementary figure 5: subgroup analysis – allergic reactions**

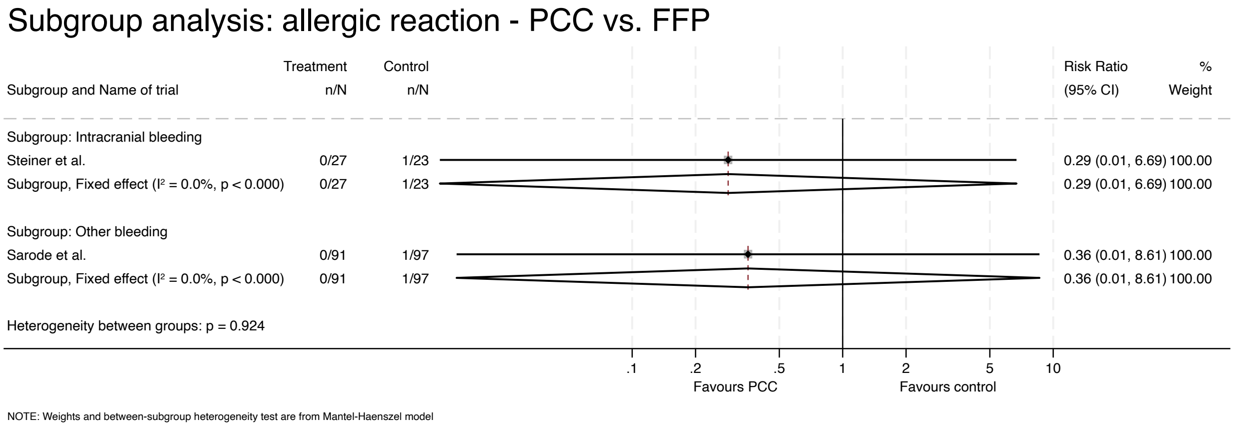


RR – relative risk, CI – confidence intervals, PCC – prothrombin complex concentrate, FFP – fresh frozen plasma

## Supplementary figure 6: subgroup analysis – tardy INR correction

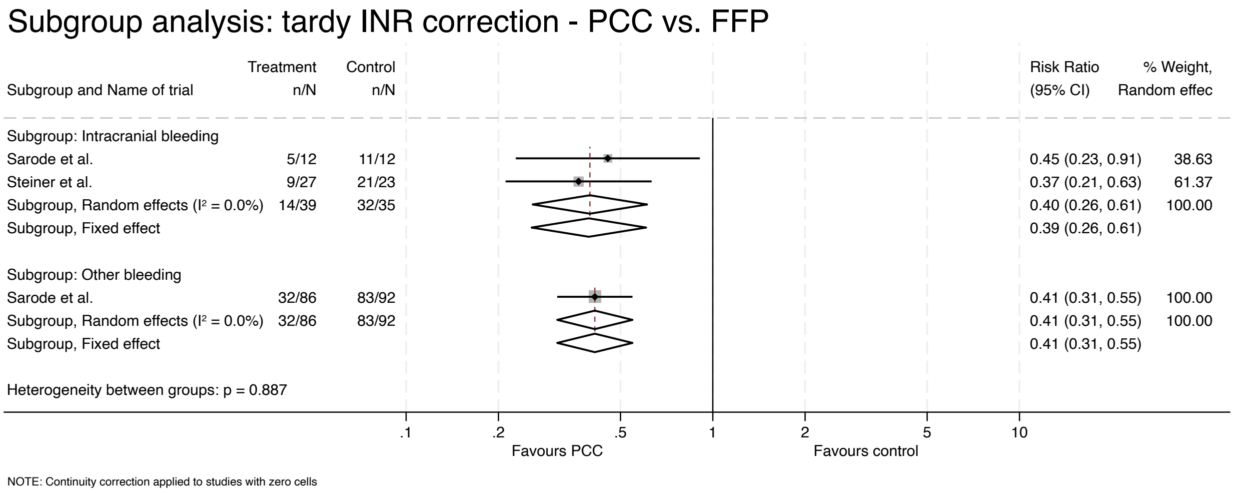


RR – relative risk, CI – confidence intervals, PCC – prothrombin complex concentrate, FFP – fresh frozen plasma

## Supplementary figure 7: subgroup analysis - poor haemostatic efficacy

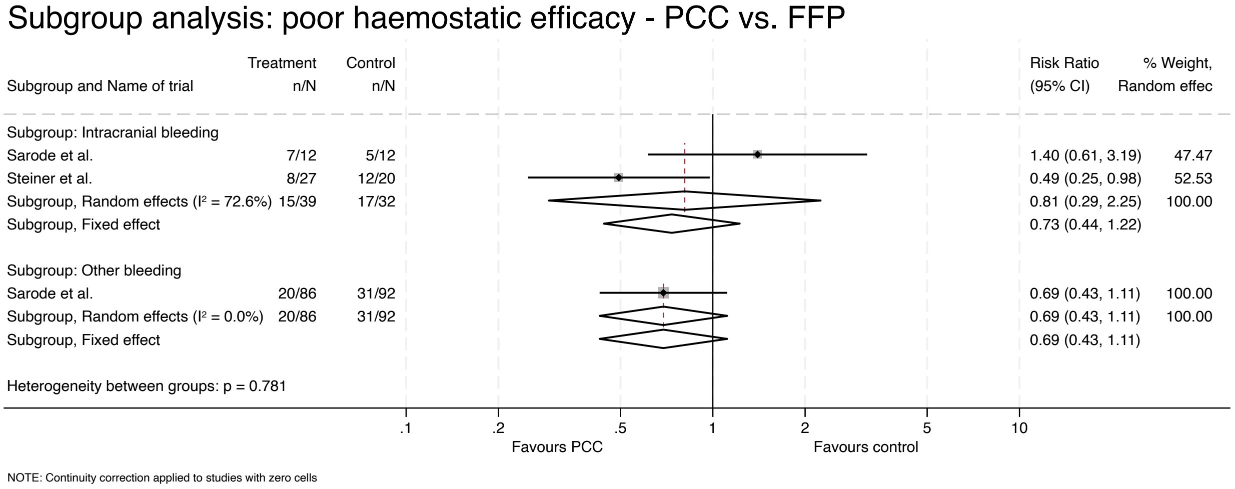


RR – relative risk, CI – confidence intervals, PCC – prothrombin complex concentrate, FFP – fresh frozen plasma
