# Supplementary material for: Prothrombin complex concentrate for reversal of oral anticoagulants in patients with oral anticoagulation-related critical bleeding: a systematic review of randomised clinical trials
Source: Scand J Trauma Resusc Emerg Med. 2025 Feb 4;33:19. doi: 10.1186/s13049-025-01334-1 (PMC11792222; doi:10.1186/s13049-025-01334-1)
Supplement: Supplementary file 11 — Additional file 11. [file 13049_2025_1334_MOESM11_ESM.pdf]

Additional file 11:

Supplement 18: Grading of Recommendations, Assessment, Development and Evaluations (GRADE) tables for each primary and secondary outcome

Supplement table 7: Summary of findings for PCC versus fresh frozen plasma for reversal of vitamin K antagonists

Outcomes	Anticipated absolute effects* (95% CI)		Relative effect (95% CI)	№ of participants (studies)	Certainty of the evidence (GRADE)	Comments
	Risk with FFP	Risk with PCC				
All-cause mortality assessed	10 per 100	10 per 100 (3 to 40)	RR 1.05 (0.27 to 4.05)	262 (2 RCTs)	⊕⊕◯◯ LOW [a]	Dichotomous unsubjective outcome making incomplete blinding less a concern.
Health-related quality of life	Mean HRQOL score 8.21	Mean HRQOL score on average 1.04 lower (95% CI -0.94 to 3.02)		34 (1 RCTs)	⊕◯◯◯ VERY LOW [a,b]	
Serious adverse events	27 per 100	36 per 100 (26 to 51)	RR 1.33 (0.94 to 1.88)	262 (2 RCTs)	⊕◯◯◯ VERY LOW [a,b]	
Poor functional outcome	50 per 100	53 per 100 (35 to 81)	RR 1.06 (0.70 to 1.62)	68 (2 RCT)	⊕◯◯◯ VERY LOW [a,b]	Only evaluated for participants with intracranial haemorrhage
Thromboembolic events	7 per 100	11 per 100 (5 to 25)	RR 1.60 (0.70 to 3.61)	262 (2 RCTs)	⊕◯◯◯ VERY LOW [a,b]	
Allergic reactions	2 per 100	0 per 100 (0 to 5)	RR 0.32 (0.03 to 2.99)	262 (2 RCTs)	⊕◯◯◯ VERY LOW [a,b]	
Pulmonary oedema	4 per 100	2 per 100 (0 to 10)	RR 0.53 (0.10 to 2.83)	262 (2 RCTs)	⊕◯◯◯ VERY LOW [a,b]	

FFP: Fresh frozen plasma; PCC: Prothrombin complex concentrate; CI: Confidence interval; RR: Risk ratio; HRQOL: Health-related quality of life.
*The risk in the intervention group (and its 95% confidence interval) is based on the assumed risk in the comparison group and the relative effect of the intervention (and its 95% CI).
a. Quality of evidence rated down two levels due to major imprecision.
b. Quality of evidence rated down one level due to major concerns regarding observer-blinding. It is unclear if the observers who detected serious adverse events, thromboembolic events, allergic reactions and pulmonary oedema were blinded to allocation.

Supplement table 8 Summary of findings for PCC plus fresh frozen plasma versus fresh frozen plasma alone for reversal of vitamin K antagonists

Outcomes	Anticipated absolute effects* (95% CI)		Relative effect (95% CI)	№ of participants (studies)	Certainty of the evidence (GRADE)	Comments
	Risk with FFP alone	Risk with PCC plus FFP				
All-cause mortality	38 per 100	25 per 100 (6 to 100)	RR 0.65 (0.16 to 2.59)	21 (1 RCT)	⊕◯◯◯ VERY LOW [a,b]	
Health-related quality of life	See comments	See comments	Not estimable	(0 RCTs)	-	No available evidence as the outcome is not evaluated
Serious adverse events	70 per 100	29 per 100 (8 to 99)	RR 0.41 (0.12 to 1.41)	17 (1 RCT)	⊕◯◯◯ VERY LOW [a,b]	
Poor functional outcome	See comments	See comments	Not estimable	(0 RCTs)	-	No available evidence as the outcome is not evaluated
Thromboembolic events	13 per 100	6 per 100 (0 to 100)	RR 0.50 (0.02 to 10.34)	13 (1 RCT)	⊕◯◯◯ VERY LOW [a,b]	
Allergic reactions	See comments	See comments	Not estimable	(0 RCTs)	-	No available evidence as the outcome is not evaluated
Pulmonary oedema	13 per 100	6 per 100 (0 to 100)	RR 0.50 (0.02 to 10.34)	13 (1 RCT)	⊕◯◯◯ VERY LOW [a,b]	

FFP: Fresh frozen plasma; PCC: Prothrombin complex concentrate; CI: Confidence interval; RR: Risk ratio
*The risk in the intervention group (and its 95% confidence interval) is based on the assumed risk in the comparison group and the relative effect of the intervention (and its 95% CI).
a. Quality of evidence rated down two levels due to major concerns about the risk of bias affecting the effect estimate.
b. Quality of evidence rated down two levels due to major imprecision.

Supplement table 9: Summary of findings for PCC plus fresh frozen plasma versus fresh frozen plasma alone for reversal of factor Xa inhibitors

Outcomes	Anticipated absolute effects* (95% CI)		Relative effect (95% CI)	№ of participants (studies)	Certainty of the evidence (GRADE)	Comments
	Risk with FFP alone	Risk with PCC				
All-cause mortality	19 per 100	15 per 100 (4 to 59)	RR 0.65 (0.16 to 2.59)	41 (1 RCT)	⊕○○○ VERY LOW [a,b]	
Health-related quality of life	See comments	See comments	Not estimable	(0 RCTs)	-	No available evidence as the outcome is not evaluated
Serious adverse events	See comments	See comments	Not estimable	(0 RCTs)	-	No available evidence as the outcome is not evaluated
Poor functional outcome	See comments	See comments	Not estimable	(0 RCTs)	-	No available evidence as the outcome is not evaluated
Thromboembolic events	0 per 100	Unable to estimate	RR 3.14 (0.14 to 72.92)	41 (1 RCT)	⊕○○○ VERY LOW [a,b]	
Allergic reactions	See comments	See comments	Not estimable	(0 RCTs)	-	No available evidence as the outcome is not evaluated
Pulmonary oedema	See comments	See comments	Not estimable	(0 RCTs)	-	No available evidence as the outcome is not evaluated

FFP: Fresh frozen plasma; PCC: Prothrombin complex concentrate; CI: Confidence interval; RR: Risk ratio
*The risk in the intervention group (and its 95% confidence interval) is based on the assumed risk in the comparison group and the relative effect of the intervention (and its 95% CI).
a. Quality of evidence rated down two levels due to major concerns about the risk of bias affecting the effect estimate.
b. Quality of evidence rated down two levels due to major imprecision.
